# Barriers and facilitators to implementing a single-visit, screen-and-treat approach with thermal ablation for cervical cancer prevention in Kenya

**DOI:** 10.1371/journal.pgph.0005166

**Published:** 2025-09-09

**Authors:** Harriet Fridah Adhiambo, Emmah Owidi, Phelix Okello, Megan Coe, Michelle B. Shin, Lynda Myra Oluoch, Nicholas B. Thuo, Valary Ihaji, Mary Bernadette Kerubo, Alex Kinyua, Jason Caucutt, Jesse Heitner, Thomas Odeny, Bryan Weiner, Kenneth Ngure, Nelly Mugo, Sarah Gimbel

**Affiliations:** 1 Department of Child, Family, and Population Health Nursing, School of Nursing, University of Washington, Seattle, Washington, United States of America; 2 Center for Microbiology Research, Kenya Medical Research Institute, Nairobi, Kenya; 3 Center for Clinical Research, Kenya Medical Research Institute, Nairobi, Kenya; 4 Department of Global Health, University of Washington, Seattle, Washington, United States of America; 5 Division of Infectious Diseases, Massachusetts General Hospital, Harvard Medical School, Boston, United States of America; 6 Department of Medicine, Washington University in St. Louis, St. Louis, Missouri, United States of America; 7 School of Public Health, Jomo Kenyatta University of Agriculture and Technology, Nairobi, Kenya; McGill University, CANADA

## Abstract

Cervical cancer continues to be a major global threat to women's health, with approximately 660,000 women diagnosed annually, 94% of whom are in low- and middle-income countries (LMICs). The high disease burden in LMICs is partly due to suboptimal adoption and widespread implementation of effective preventive interventions. This study explored drivers of implementation success and failure for a future single-visit, screen, and treat approach with thermal ablation (SV-SAT + TA), referred to as TIBA in Kenya. Guided by the Consolidated Framework for Implementation Research (CFIR framework), we conducted in-depth interviews with 34 participants (frontline health workers, health facility managers, and policymakers) between May and August 2022 in Kiambu, Embu, and Murang'a Counties in Kenya. All interviews were audio recorded and transcribed verbatim. We applied deductive and inductive coding for emerging themes. The participants reported the relative advantage of thermal ablation for the single-visit, screen-and-treat approach, emphasizing its lower start-up and maintenance costs and lower complexity compared to cryotherapy. Additionally, participants expressed confidence in their ability to implement TIBA, and a strong commitment from the leadership to support TIBA implementation was reported. These factors were perceived as drivers of successful TIBA implementation. In contrast, barriers, including lack of essential commodities and equipment, shortage of trained providers, staff redeployment, inadequate space, recruitment challenges, and silos within the healthcare system, were identified as drivers of implementation failure. To optimize cervical cancer prevention efforts in LMICs, it is critical to address both systemic and contextual factors through a coordinated, integrated, and system-wide approach that involves all the key stakeholders.

## Introduction

Cervical cancer remains a major global threat to women's health, ranking as the fourth most common cause of cancer among women [1]. The World Health Organization (WHO) estimates that globally, approximately 660,000 women are diagnosed with cervical cancer, with about 350,000 deaths reported annually [2,3]. Women in low- and middle-income countries (LMICs) account for 94% of the disease burden [4]. In 2022, cervical cancer contributed to 12% of all cancer deaths in Kenya [5]. Despite the availability of preventive interventions such as the human papillomavirus (HPV) vaccination and routine screening and treatment of precancerous cervical lesions and early cancer [6], their availability, affordability, and uptake remain limited in many LMICs. This is due to structural, operational, and behavioral barriers constraining providers’ and patients’ access to and utilization of services [7,8]. Addressing these challenges requires a deeper understanding of implementation determinants to improve access and uptake of these preventive interventions, ultimately reducing the global burden of cervical cancer.

The standard practice for cervical cancer screening in LMICs, including Kenya, involves a single-visit, screen-and-treat approach (SV-SAT) [9–11]. This strategy utilizes visual inspection with acetic acid (VIA) for screening, followed by immediate treatment of screen-positive lesions with either cryotherapy or loop electrosurgical excision procedure (LEEP), a method referred to as SV-SAT+Cryotherapy or LEEP [12]. The single-visit approach requires fewer resources compared to multistep processes such as screening with VIA or HPV DNA testing followed by a diagnostic evaluation (e.g., colposcopy or biopsy) and subsequent treatment. It enables broader population coverage and provides immediate treatment, reducing the need for repeat visits and minimizing loss to follow-up [13]. Since 2013, Kenya has adopted the SV-SAT+Cryotherapy or LEEP for eligible women aged 25–49 years [14] as outlined in the national guidelines. However, the availability of screening and treatment services remains limited, with substantial facility-level disparities. Screening is predominantly opportunistic in Kenya [15], and the SV-SAT+Cryotherapy strategy has resulted in low service uptake, with approximately 16% of eligible women screened and 22–39% of women diagnosed with precancerous lesions receiving treatment [16–18]. These figures fall far short of the ambitious WHO target of 70% of women screened by 35 years of age, and again, at 45 years, and 90% of women with precancerous lesions treated [7,19]. The low screening uptake in Kenya is attributed to factors such as lack of awareness, social influence, such as health care decisions made by partners, fear, high provider workload, lack of trained staff, limited availability of screening services, and insufficient essential supplies, including gloves and acetic acid [20,21]. The low treatment compliance is primarily associated with logistical and supply chain challenges, specifically the inconsistent supply of nitrous oxide, a critical component in cryotherapy treatment [17]. Additionally, the number of providers trained in LEEP is limited and concentrated in higher-level facilities, which has further hindered its effective use to increase treatment [22,23]. These challenges have led to missed opportunities for screening, losses to follow-up, and incomplete treatment. As a result, 90% of women diagnosed with cervical cancer (approximately 5236 diagnosed annually) in Kenya present at an advanced stage [24,25]. Recommended by the WHO as an alternative treatment for precancerous lesions [26], thermal ablation (TA) provides a promising alternative to bridging the current gaps in the single-visit screen-and-treat initiatives for cervical cancer prevention in LMICs. In Kenya, TA is part of the cervical precancer treatment interventions [27]. Compared to cryotherapy, TA is portable, may have lower infrastructural challenges and maintenance costs, and is better suited for primary care settings [24,28].

Kenya is preparing for a national rollout of the SV-SAT + TA to enhance population-based cervical cancer screening and treatment. As part of this effort, we implemented a stepped-wedge, cluster-randomized trial that aims to develop and test implementation strategies to inform the nationwide scale of the SV-SAT + TA. We will use “TIBA,” a Swahili word for treatment, to represent the SV-SAT + TA. Prior to the rollout, we conducted formative work with implementers (frontline healthcare providers and health facility managers) and policymakers to gather their perspectives on factors that could promote or hinder successful TIBA implementation. Understanding these implementation determinants at multiple levels is critical for effective planning, resource optimization, and achieving national cervical cancer prevention goals. These findings will provide actionable insights for the Kenyan Ministry of Health (MOH) and offer applicable lessons to other LMICs aiming to implement similar strategies, contributing to the global efforts to eliminate cervical cancer.

## Methods

### Ethics statement

This study was approved by the Scientific and Ethics Review Unit of Kenya Medical Research Institute (SERU-KEMRI No. 4403) in Nairobi, Kenya, and the Human Subjects Institutional Review Board (STUDY00014200) at the University of Washington, Seattle, USA. All participants provided written consent to participate in interviews.

### Setting

#### Kenyan health system.

The healthcare system in Kenya provides services across six levels of facilities. The county manages levels I-V, while level VI is managed at the national level [29,30]. Apart from government-led health facilities, Kenya's health systems also include faith-based health facilities, which contribute approximately 30% of health services [31]. They are overseen by faith-based organizations and health facilities operated with support from non-governmental organizations (NGOs). Cervical cancer screening across these levels is primarily opportunistic, occurring at reproductive health clinics and outpatient clinics. Complicated cases, such as suspicious cases for cancer or precancer treatment requiring LEEP services, are referred to higher-level facilities with the necessary equipment and trained personnel.

#### Study setting.

**Fig 1:**
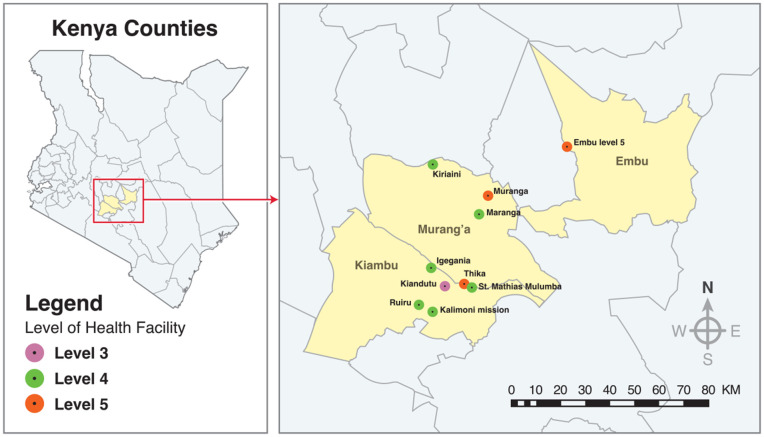
Map of Kenya showing the distribution of TIBA health facilities.

This study purposively sampled ten facilities across three levels (III, IV, V), including public and faith-based-managed facilities with varying levels of support from partner organizations. The study took place in Kiambu, Embu, and Murang'a counties in Kenya, which serve an estimated 4.1 million inhabitants (Fig 1). The heterogeneity of facilities engaged in the TIBA study (Table 1) provides a robust representativeness of the healthcare landscape in Kenya, bolstering the potential generalizability of our research findings and supporting the development of a scalable TIBA model for Kenya and other LMICs.

**Table 1:** Health facility characteristics.

County and Health Facility Name	Health Facility Level			Management		Average monthly outpatient visits
	V	IV	III	Government	Faith-based	
**Kiambu County**						
Thika	√			√		58,458
Igegania		√		√		5,137
St Matias Mulumba		√			√	6,292
Ruiru		√		√		19,712
Kiandutu		√		√		2,489
Kalimoni Mission		√			√	5,551
**Embu County**						
Embu	√			√		37,331
**Murang’a County**						
Maragua		√		√		18,074
Murang’a	√			√		33,315
Kiriaini Mission			√		√	14,296

Source (Health facility records department and the Kenya Master Health Facility Registry).

Table 1 above shows characteristics of participating clinics in the study across the three counties.

### The TIBA strategy

**Fig 2:**
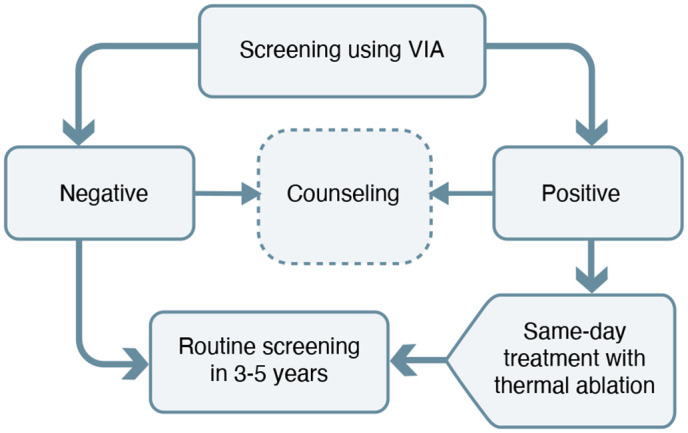
TIBA strategy.

TIBA (Fig 2) is a dual-pronged implementation strategy targeting women of reproductive age (25–49 years) that consists of two steps:

1) Screening of the cervix using visual inspection with acetic acid (VIA).
2) Same-day treatment using thermal ablation.

TIBA is introduced to facility staff over six months with support from county-level advisors, and the target implementers are healthcare facility staff, including reproductive health clinic nurses (primary implementers), clinical officers, and medical officers [12].

### Study design

This cross-sectional descriptive qualitative study applied in-depth interviews to collect data before the TIBA rollout. This study design provides an overview of the current situation while enabling the collection of contextually rich data on participant perspectives and experiences. It prioritizes stakeholder engagement, which is critical for facilitating intervention adoption and sustainability, thereby increasing the likelihood of successful implementation and scale-up.

### Sampling and participant recruitment

Thirty nurses, clinical officers, and medical doctors were purposively sampled from 10 healthcare facilities across the three counties. Two categories of providers were recruited: frontline healthcare providers (n = 20) and health facility managers(n = 10). Four national-level public-sector policymakers from the Ministry of Health, the National Cancer Institute of Kenya, the National Cervical Cancer Program, the Kenyatta National Teaching Hospital oncology unit, and a Kenya Medical Education Trust representative were interviewed. Each of these individuals plays a critical role in influencing policy decisions on cervical cancer prevention and treatment nationally. Multiple strategies for participant recruitment were employed, including email outreach, phone calls, and in-person recruitment at the study facilities.

It is important to note that prior to this formative research, seven out of ten health facilities had been exposed to thermal ablation through training by other implementing partners. However, only two of these facilities actively applied thermal ablation in practice. As a result, only participants from these two health facilities had firsthand experience with thermal ablation, while others did not.

### Theoretical framework

The adapted Consolidated Framework for Implementation Research (CFIR) for LMICs [32] informed the development of semi-structured interview guides used to explore end users’ (clinicians) and policymakers’ perspectives on potential drivers of TIBA's future implementation success or failure. CFIR was selected for its pragmatic and proven approach to investigating barriers and facilitators and its successful application across diverse settings, making it highly relevant for this study [33,34].

#### Identification of CFIR domains and constructs.

We began by mapping our intervention to the CFIR framework. During team discussions supported by review of the literature and investigators’ knowledge and experience implementing similar strategies in Kenya and regionally, we identified and prioritized 16 constructs across the five CFIR domains, which were included in the question guide (S1 Table). These constructs were: relative advantage, adaptability, complexity, evidence strength and quality, health system context, policies and guidelines, structural characteristics, networks and communication, implementation climate, readiness for implementation, knowledge and beliefs about the intervention, self-efficacy, individual stage of change, and patients’ needs and resources. We pilot-tested the interview guide with three potential end-users from health facilities and one representative from the policy realm. We used their feedback to refine the interview questions and probe structure.

### Data collection

Four qualitative research assistants (authors EO, PO, VO, and NBT) conducted the interviews. Each assistant had more than five years of experience conducting qualitative research with women of childbearing age in Central Kenya. They conducted in-depth interviews with 20 healthcare providers and nine health facility managers between May and August 2022. Twenty-nine interviews were conducted in private rooms within the health facilities, and five were done on Zoom due to scheduling constraints. Each interview lasted between 45–60 minutes and was conducted in English. Participants were reimbursed Ksh1000 (~ 8 USD) for transport costs; otherwise, no monetary fees were paid to participants. All interviews were audio recorded and transcribed verbatim. Thematic saturation was achieved with the planned number of participants across categories [35,36].

### Analysis

#### Codebook development.

A priori codes were drafted to capture the selected CFIR constructs [37]. Our four-member team (authors, NBT, HAF, EO, and PO) translated each construct into operational definitions and included exemplar quotes. For example, “Relative advantage” was defined as any text where the health worker or health facility manager discusses their perception of the advantage of implementing thermal ablation over cryotherapy or LEEP for managing precancerous lesions. was developed and used by a four-member team to code transcripts.

#### Application of the coding scheme.

Initially, a four-member team concurrently applied deductive coding to the same three transcripts: one each from a frontline healthcare provider, a health facility manager, and a policymaker using Dedoose software [38] to assess the adequacy of the codebook.. There are a few codes that were inductively added. A series of six consensus meetings were held to review and discuss the coding results and make necessary refinements to the codebook. The final, refined codebook was used by a three-member team (authors HAF, EO, and PO), who incorporated identified complementary field notes to complete the coding of the remaining 31 transcripts.

#### Thematic analysis.

Once coding was complete, we exported the excerpts and code reports to examine patterns and themes related to the constructs. We synthesized these patterns into overarching themes organized by the CFIR constructs, thereby aligning our analysis in the theoretical framework. We structured our findings around each construct within the relevant domains, facilitating comparisons on how each construct influenced TIBA implementation.

It is important to note that the analysis of the CFIR intervention domain compared thermal ablation to cryotherapy, and the other domains considered TIBA, i.e., the single-visit, screen, and treat approach with thermal ablation (SV-SAT + TA). This is because thermal ablation was the most novel component of the intervention from the participant's perspective. While the broader SV-SAT model was already familiar to many health workers, having been previously implemented using cryotherapy, thermal ablation was relatively new in this setting and still in the early stages of introduction. The unit of analysis was the health system level (national versus health facility). In addition, management (public sector versus faith-based) and size (< 3000 monthly visits = smaller health facilities, > 5000–17999 = moderately sized health facilities, and >18,000 monthly visits = larger) were considered as potential site-specific factors influencing TIBA implementation.

## Results

### Study participants

Thirty-four participants were interviewed: nine from level V, 19 from level IV, and two from level III facilities. The majority of participants were nurses, n = 23. (Table 2).

**Table 2:** Participant characteristics.

Cadre	Participants	
	Manager (n = 14)	Provider (n = 20)
Nurse	4	19
Clinical Officer	1	0
Medical Officer	2	0
Obstetrician and Gynecologist	3	1
Policymakers	4	0

Table 2 above shows participant characteristics.

The CFIR domains of innovation characteristics and inner settings were most often cited as driving future TIBA implementation success or failure. Within these two domains, five CFIR constructs, cost, complexity, relative advantage, leadership engagement, and self-efficacy, emerged as greater drivers of successful TIBA implementation across health system levels. In contrast, constructs on available resources, structural characteristics, and health system context were identified as significant barriers. Additionally, constructs on policies, programs, and guidelines were reported to negatively and positively influence future TIBA implementation. (S2 Table)

### Intervention characteristics-complexity, cost, and relative advantage

#### National level.

Policymakers felt thermal ablation was user-friendly and provided a relative advantage over cryotherapy due to its portability and lower setup and maintenance costs (TA does not require regular consumables). They also highlighted that TA, being chargeable in multiple ways, can treat more than 20 patients without the need to recharge. Its portability was reported to be suitable for outreach activities to communities with limited accessibility. These were perceived as facilitators for the successful future implementation of TIBA.

*It is easier and less complex compared to cryotherapy. It does not require regular filling of the consumables. It's portable and can potentially improve linkage to care (Policymaker, 003).*

Despite these advantages, a policymaker stated that maintaining TA may be a potential challenge because hospital maintenance staff lack experience servicing the TA machines and, thus far, have not received this training.

*There is a need to orient the biomedical maintenance unit in the health facility, as they don't seem to understand the thermal ablator. (Policymaker, 002)*

#### Health facility-level.

Providers and health facility managers working across facilities reinforced the national level's belief that thermal ablation was easier and thus offered a comparative advantage over cryotherapy. Specifically, informants reported that the TA machine was easy to assemble and time-efficient, requiring 30 seconds for treatment compared to approximately 10 minutes with cryotherapy. They reported other benefits such as portability, reduced storage space needs, fewer logistical and supply chain challenges, and its potential to minimize client distress because treatment could be completed more quickly relative to cryotherapy. Providers and managers felt that it may be less costly to operate TA as it only requires batteries or solar, compared to regularly purchasing gas for cryotherapy.

*It doesn't have many steps, and it's not complicated. Doing the treatment is not hard because it's just connecting the machine, and you do it. (Nurse, 016)*

### Inner setting: Structural characteristics

#### National level.

As a facilitator, the policymakers highlighted that each sub-county has trainers for cervical cancer screening and treatment who offer mentorship to providers to curb challenges with staff turnover. Additionally, cervical cancer screening and treatment sessions are included during the health facilities’ continuous medical education (CMEs). These trainers and CMEs are key to TIBA's successful future adoption and implementation.

*So, for the first entry, we usually train a minimum of two. We also have mentors who are trainers in clusters at the sub-county level. (Policymaker, 002)*

#### Health facility-level.

Unlike at the national level, where structural supports were described as being in place, the health facilities identified current structural issues as significant barriers to future TIBA implementation. Some providers (primarily nurses) across the ten health facilities were trained in cervical cancer screening and TA treatment, and five facilities received donated TA machines and screening supplies, yet screening and treatment rates were low, as reported by providers. Inadequate space (commonly cited in smaller facilities) was highlighted as a significant challenge, resulting in long queues and compromised privacy and confidentiality for clients seeking services at integrated service delivery points. In larger level IV & V facilities with multiple screening points, some had only one TA machine, limiting clients and reducing overall treatment numbers.

*Our rooms are tiny, the ones the partners had the structure for the CCC (HIV treatment rooms. (Clinical Officer, 009)*

*It's hard for us now to link the screening to the treatment area because of the different service points. So, the screening can be done, but the treatment may be delayed or may not be done at all. (Obstetrician-Gynaecologist,003)*

Additionally, existing challenges such as shortage of trained staff, high staff turnover, and the perception of increased workload with TA were noted as challenges to future TIBA implementation by providers and managers across the three facility levels. Larger-level health facilities were more impacted by staff turnover relative to smaller sites. In response to these existing challenges that would potentially influence future TIBA implementation, respondents recommended regular, routine provider training across cadres, along with ongoing mentorship to address these issues. One respondent proposed designating specific days each week for cervical cancer services to better align care delivery staff workload and reduce operational strain.

*I'm the only nurse offering services. Right now, even with VIA VILI, sometimes I can't do it because I'm alone, the queue is very long, and we have a high workload (Nurse, 006).*

*If we can allocate a specific day, we set aside and say that day is for cervical cancer screening and treatment, it will be effective and more efficient (Nurse, 001).*

### Readiness for implementation: Leadership engagement and available resources

#### National level.

National-level respondents felt the Kenyan health system was ready to implement TIBA. Readiness was attributed to the availability of expertise nationwide to support protocol implementation and national health leadership's expressed commitment to reviewing reproductive health policies and advocating for health financing for women's health.

*I think they [policymakers] are well versed that they can advise and guide on developing appropriate guidelines. The policies are already there for screening, and there is relevant expertise to be able to guide that process (Policymaker, 004)*

As noted by national-level respondents, the available resources to support future TIBA included training and commodities. Cervical cancer screening and treatment training for health providers had been carried out in half of the counties (22 counties out of 47) nationwide, and partnerships exist with multilateral organizations such as the Global Fund to purchase cervical cancer-related commodities and provide cervical cancer training for the remaining half of counties in Kenya. Additionally, policymakers noted that cervical cancer prevention activities had been incorporated into existing program planning, such as family planning and HIV services, to improve efficiency and expand access. Finally, workflow algorithms, information, education, and communication (IEC) materials, and guidelines were reported to have been developed by the Ministry of Health at the national level. These inputs were perceived as facilitators to effectively implementing TIBA.

*I am happy to report that we partnered with the Global Fund. As we speak, we've already integrated a lot of cervical cancer commodities, such as speculums, in this current cycle. We will also train another 22 counties using Global Fund's support (Policymaker, 001).*

Human resource constraints are a major challenge, especially at the county level. In reference to this current barrier, national-level respondents proposed collaborations with various implementing partners to support hiring additional staff, with the caveat that leveraging donor support was ultimately unsustainable.

*We know that counties have human resource challenges. If any partners can bring in additional personnel to support that service, that will be welcomed, although we don't promote it because it is not sustainable (Policymaker, 001).*

Engaging political stakeholders was identified as necessary for securing more resources to expand cervical cancer screening and treatment nationally. However, this would require effective packaging and dissemination of information to politicians. Ensuring their support requires convincing them of the immense need for these preventive interventions for women and the feasibility of effectively reallocating resources to this area.

*A lot of strengthening is needed on the political side of policymaking to create the connection between this intervention and the ultimate goal of averting cervical cancer. Reframing the benefits of this intervention from a political perspective can ensure prioritization and resource allocation. (Policymaker, 003)*

#### Health facility-level.

Most health facility managers reported a strong commitment to implement TIBA with the provided inputs of training, CMEs, and essential commodities for cervical cancer screening and treatment in partnership with the County government and donors. Their readiness was influenced by previous experiences implementing new tools and approaches.

*Well, I am very prepared. I have been in management for thirteen years, so trust me. We are prepared. (Clinical Officer, 005).*

However, one manager from a larger facility felt ill-prepared to implement TIBA due to the current shortages of trained staff and consumables, inadequate space, coupled with lack of support from the administration. The lack of support was linked to management's inability to provide resources for cervical cancer activities such as outreaches. The manager noted that these challenges have not yet been addressed, even with the use of cryotherapy, which had been in place before the introduction of thermal ablation.

*We have done many things, but without the administration's support, these projects always fail. I have even tried doing outreaches in this county in the past, but then they died because of a lack of support. (Obstetrician-Gynaecologist, 006)*

Most health providers, however, felt confident in their leadership's support and benefitted from implementing partners’ support for other reproductive health initiatives to ensure the successful implementation of new approaches. However, there was a word of caution from providers and managers regarding the need to train individuals engaged in service delivery rather than just managers. Policymakers also shared these concerns. In addition, providers noted that supportive supervision by the leadership was deemed crucial to enhancing provider competency and confidence.

*So currently, in the county, they are supportive of the treatment of precancerous lesions. They are partnering with different donors so that we can increase the screening of women in reproductive health clinics. (Nurse, 005).*

*Mostly, they train people who never see clients. For training and refresher courses, let them consider training people on the ground who review patients. Also, continuous on-the-job mentorship, supervision, and meetings are necessary to identify gaps and determine progress (Nurse, 009).*

Regarding available resources, providers and managers emphasized that their readiness to implement TIBA was tied to addressing the current facilities’ pragmatic gaps in human resources, equipment, and other essential commodities such as gloves, speculums, and acetic acid. If these barriers were not addressed, successful future implementation of TIBA would likely be stymied. Forecasting, budgeting, and including these needs in annual work plans were identified as ways to ensure continuity in service provision.

*We need refresher training, the actual machine (TA), supportive supervision, and mentorship from the national government. If we can get staff employed, this will address the staff shortage (Nurse, 004).*

*When doing the annual work plan, they can incorporate a budget for cancer screening, training, etc. It is possible and doable with proper planning (Nurse, 007).*

### Characteristics of systems: Health system context

#### National level.

Policymakers identified human resources, insufficient funding for cervical cancer preventive services, supply chain management, and infrastructure as the main health system factors that would hinder the successful implementation of TIBA. To achieve this, they proposed reducing donor dependency and ensuring adequate budget allocations for cervical cancer prevention activities at the County level.

*One is the buy-in and changing that attitude from funder- or partner-driven to county-led and county-driven. So, there is a lot of work that we need to do to ensure that the county budgets for this and that it is not seen as a partner initiative. (Policymaker, 002* **)**

#### Health facility-level.

Providers and managers, like the policymakers at the national level, identified human resources, logistical and supply challenges, and essential commodities for cervical cancer screening and treatment as key factors within the health system that would hinder the successful execution of TIBA. These challenges included frequent staff transfers across health facilities without immediate replacement, staff changeovers, and lengthy procurement processes contributing to service interruptions.

*So, the procurement, I said, is a long process. When our gas [for cryotherapy] went off, we stayed for six months, and one of the partners bought for us that gas, the one that we are using currently. (Nurse, 007)*

*You find that the county has decided to reassign staff from here, affecting the implementation of services. You might introduce something, and we pick it, then in between, things fall apart with the staff changeover. I'm left now with nobody trained. (Nurse, 008)*

Other factors within the health system reported by providers and managers that may influence TIBA implementation negatively were unsupportive provider attitudes. Unsympathetic provider attitudes were reported to stem from work burnout, lack of motivation because of delayed salaries, and overall lack of workplace resources. These complaints were common, especially among providers in larger health facilities.

*I have noted that most healthcare workers have negative attitudes toward the screening. I think it is more from burnout, shortage of workers, being demotivated, and lack of resources. (Clinical Officer, 005)*

*Staff demotivation is at an all-time high; you find people staying for so long without salaries, even two months, and you expect them to work. (Obstetrician-Gynaecologist, 006)*

Faith-based facilities (more so than public facilities) highlighted challenges with securing agreement from patients to screen and treat for cervical cancer. These facilities charge more for screening and treatment, a deterrent for many lower-income women in the community. To address this challenge, faith-based facilities offered free educational outreach activities to create awareness and encourage women to attend screening and treatment of precancerous cervical lesions at their facilities. In contrast, public sector sites focus more on in-reach activities (e.g., recruiting from other departments within the same facility), as outreach in the communities poses logistical and financial challenges.

*There's a service cost for screening and treatment, affecting TIBA implementation. If the person has no money to pay, they will not receive the service. (Nurse, 014-Faith-based health facility)*

*Actually, in-reaches are less expensive than outreaches because you must be facilitated to go out. (Nurse, 002-Public health facility)*

### Policies, programs, and guidelines

#### National level.

Existing policies, guidelines, and protocols on cervical cancer screening and treatment were recognized as facilitating implementation. They provided a structured framework for cervical cancer screening and treatment implementation, including strategies like TIBA, aligning with WHO-recommended best practices. Two policymakers acknowledged that HPV testing is the gold standard for cervical cancer screening. However, Kenya is widely implementing VIA due to limited resources to implement HPV testing, which requires access to an expensive DNA testing platform. Existing DNA testing capacity in Kenya is primarily from GeneXpert machines acquired through and prioritized for use by tuberculosis screening programs.

*We have national cancer screening guidelines in place which serve to guide all stakeholders on screening and treatment (Policymaker, 001)*

*What is recommended by these screening guidelines is HPV (Human Papilloma Virus) DNA testing. We advocate for VIA because resources for pathology are limited. (Policymaker, 003)*

Despite the existing policies and guidelines, gaps in understanding how patients navigate referral pathways and follow-up procedures persist. These gaps lead to inefficiencies in service provision and missed opportunities for timely care and treatment, impeding progress toward cervical cancer elimination.

*Implementing population-based screening may be challenging because, despite having screening guidelines, there is no system to track which patients complete screening, follow up on results, or return for care. (Policymaker, 004).*

#### Health facility-level.

Existing programs supported by implementing partners were seen as potentially helpful to future TIBA implementation. These programs increased access to cervical cancer services by providing the facilities with screening supplies, training, equipment, and supportive supervision. This was common across health facility levels.

*We have had so many programs that the partners have been supporting, and they have been of great help because we have gone around to most of the periphery facilities to screen and try to mentor the ones there. (Nurse, 007)*

Cervical cancer screening and treatment guidelines were available onsite at all health facilities, except for two larger facilities with old and outdated versions. It was unclear why they did not have access to the updated version.

*We have a cervical cancer screening guideline that we are using, and that is what I am saying. I am not sure if it is 2016 or 2014, and you know things have changed. We should have an updated one. (Nurse, 004)*

A concern voiced by smaller health facilities regarding existing cervical cancer programs was the variation in program priorities among the implementing partners who provided cervical cancer screening and treatment supplies. For example, some partner-supported programs only allowed services for HIV-infected women. Staff felt this caused unnecessary and unethical disparities in access to services, was an inefficient use of resources, and would undermine the effective implementation of TIBA. Additionally, health facility managers reported that variable remuneration by program (HIV or non-HIV-specific) and county government for nursing staff could potentially negatively impact TIBA implementation and cause staff demotivation because they perform similar activities but are paid differently.

*Screening can be a bit tricky because the organization that is giving us the materials specifically provides for women living with HIV only. (Nurse, 013)*

*The nurse working in the CCC will deal with her patients, and those on the other side [regular clinics like MCH] will feel demotivated because they know the ones in the program are being paid more. Services need integration, whether they are supported programs or not. (Nurse, 008)*

### Individual characteristics: Self-efficacy

#### Health facility-level.

Most health facility providers and managers across sites expressed confidence in their ability to implement TIBA. This confidence was influenced by several key factors, including previous experience with cervical cancer screening and treatment (e.g., cryotherapy), support from management, knowledge, and skills related to thermal ablation, and a track record of successfully implementing other healthcare interventions. However, a minority of managers (n = 2) reported less confidence because of limited knowledge and skills related to thermal ablation. These individuals also noted that staff were still learning and felt uneasy about how patients and staff would receive the intervention. One of them suggested that with good training, they would be confident in delivering TIBA instead of referring clients to larger sites (standard practice).

*I would not say I am very confident. You know it is a new thing. So, we are learning and eager to learn, but I cannot predict how the patients and health workers will take it, so I am eagerly waiting. (Obstetrician-Gynaecologist 010)*

## Discussion

Our study identified nine key CFIR constructs frequently mentioned as the most influential drivers of TIBA implementation. Five constructs: cost, complexity, relative advantage, self-efficacy, and leadership engagement emerged as drivers of successful implementation. Previous research on cervical cancer screening and treatment programs with same-day screening using VIA and treatment with TA have demonstrated its suitability for low-resource settings, highlighting provider acceptability of TA due to its portability, ease of use, and affordability, which aligns with the positive reception of TIBA, as reported by the health providers, managers, and policymakers in our study [24,39,40]. A study examining healthcare workers’ experiences implementing screening and treatment for cervical cancer prevention in Malawi identified relative advantage, cost, and complexity as key positive influences for using TA [41]. However, unlike our study, where leadership engagement was perceived as a strong positive driver of TIBA implementation, Moucheraud et al. reported it as a mixed factor with both weak positive and negative influences on TIBA implementation [41]. As a building block of the WHO health system framework [42], leadership plays a critical role in facilitating or hindering the implementation of new healthcare interventions. It may vary depending on the local context or structure of the healthcare system. Strong leadership engagement can drive motivation, mobilize and allocate resources, and ensure strategic alignment with organizational goals [43].

Our study adds evidence to the existing literature on barriers to implementing the TIBA strategy by identifying key CFIR constructs. We found available resources, structural characteristics, health system context, and gaps in policies, programs, and guidelines as key barriers to implementing TIBA. These findings align with recent systematic reviews conducted on barriers to the implementation of cervical cancer preventive services in LMICs [23,39,40].

A key finding in our study was the disconnect between the national and health facility-level perceptions of readiness to implement TIBA. The national-level respondents reported positive developments related to the CFIR constructs on the availability of resources and structural characteristics. These included comprehensive provider training conducted on cancer screening and treatment, incorporation of cervical cancer activities into the existing programs to improve efficiency and access, and the availability of information, education, and communication (IEC) materials. In contrast, the health facility level participants continued to face significant challenges to successful implementation driven by shortages of trained staff, inadequate space, lack of equipment and essential commodities such as gloves, speculums, and acetic acid, and service integration challenges hindering access to cancer preventive services. This disconnect across system levels can lead to compromised care. Our findings mirror those of Paine et al., who used the Viable Systems Model (VSM) to explore health system disconnect in a specialist clinical service in New Zealand [44]. To address these issues, it is imperative to align strategic goals with the realities on the ground. Focused strategies such as cross-level conversations, implementation monitoring and evaluation, and equitable access to resources are necessary to ensure national-level priorities are effectively implemented at the sub-national (county) and facility levels.

The construct of health system architecture, specifically related to workforce gaps, procurement delays, and limited funding for cervical cancer preventive services, was a strong barrier to the effective implementation of TIBA. Participants in our study reported frequent staff changes without timely replacement, which led to disruptions in service delivery. Human resources represent a key health system pillar that is critical to delivering high-quality healthcare and achieving sustainable development goals [45,46]. Staff redeployment affects healthcare system functioning and subsequently impacts patient care through loss of investment in human capital, reduced quality of care, and increased burden on remaining staff [47,48]. The TIBA strategy relies heavily on skilled providers for screening (VIA) and treatment (thermal ablation); any personnel changes compromise timely and effective care. Healthcare organizations invest heavily in training to ensure a competent workforce for optimal patient care. Staff redeployment loses the value of the training investment in skill development, resulting in compromised care. Ministries of Health can address these gaps by collaborating with multiple stakeholders to conduct skill assessments that match the right staff to the right position before redeployment. In addition, onboarding and ongoing training targeting skill development for redeployed staff may help maintain competence across provider cadres in dynamic health systems [47,49]. Respondents also reported that lengthy and unpredictable procurement processes contributed to shortages of essential commodities for cervical cancer screening, including gloves and acetic acid. Implementing TIBA is stymied if commodities necessary for screening are unavailable. Health facility managers must prioritize the inclusion of cervical cancer commodities in annual budgetary planning and simultaneously strengthen procurement processes by supporting evidence-based forecasting of demand for screening and treatment supplies. Strengthening the internal tracking systems to monitor stock levels can help prevent shortages [50]. Notably, these challenges in human resources, inadequate funding, and logistical and supply chain highlighted above are not unique to Kenya; they have also been reported in similar contexts such as Rwanda, Nigeria, Ethiopia, and Malawi [39,50–52].

Client recruitment for cervical cancer screening and treatment at the facility level was identified as a barrier to effective TIBA implementation, specifically in faith-based health facilities. Faith-based healthcare facilities and public health facilities recruitment varied, with faith-based facilities excelling in outreach efforts in the community. In contrast, public health facilities attracted more clients through routine site-level service provision and in-reach activities (recruitment from hospital wards or other service areas). Fewer women sought care at faith-based health facilities because they 1) charged higher fees for cervical cancer screening and treatment and 2) did not offer family planning services (a key entry point for cervical cancer screening at public sector facilities). Although previous studies have reported that faith-based facilities have higher patient satisfaction with services and are perceived to provide quality care, these organizations often rely on user fees because they lack financial support from the government [53–56]. In contrast, public hospitals serve a larger proportion of their communities, especially socioeconomically disadvantaged individuals, as services are more affordable and accessible [57]. Better coordination and service sharing across faith-based and public health facilities could support the successful implementation of TIBA. For example, leveraging the outreach recruitment capabilities of faith-based health facilities with the improved accessibility and cost of treatment at public facilities could create a synergistic system whose sum is greater than its parts. Collaborative planning and communication between the management of facilities and resource sharing could support integrated service delivery and ensure a broader and more sustainable continuum of cervical cancer screening and treatment.

Significant progress has been made over the years to combat HIV, cervical cancer, maternal and infant morbidity, and mortality with support from implementing partners and donors. However, silos have been created through these efforts, which have weakened the entire healthcare system [58,59]. Participants in our study reported the need to balance program priorities driven by differing implementing partners. This lack of donor coordination was noted as an obstacle to effectively implementing TIBA. Participants highlighted negative sequelae from these siloes, including that some implementing partners only provided cervical cancer commodities for HIV-infected women and health worker discontent and demotivation was driven by pay differentials between program staff (who received supplements from implementing partners) and public health facility staff. Siloes within the health systems pose barriers to quality care and result in inefficient use of limited resources [58,60]. Barr et al. similarly observed inefficiencies in the health system in Sierra Leone, attributing them to partner- and donor-driven vertical /siloed programs that operate in isolation from the broader health system [61]. To achieve optimal outcomes in implementing cervical cancer prevention strategies like TIBA, collaborative efforts between the MOH and implementing partners are necessary to ensure goal alignment. By adopting a more integrated, system-wide approach, stakeholders can avoid the creation of such inefficiencies within the health system and ensure equitable and efficient use of resources for sustainable cervical cancer prevention efforts.

Table 3 below summarizes how these key findings impact various aspects of TIBA implementation and offers guidance for other settings planning to adopt the strategy.

**Table 3:** Summary of key findings and their implications for TIBA.

	Key Findings	Implications for TIBA implementation	Guidance for other LMIC settings
1	Relative advantage, cost, and complexity of thermal ablation	The relative advantages of thermal ablation, simplicity, and the potential for low maintenance requirements support its adoption.	Consider the nationwide rollout of TA to overcome logistical challenges associated with cryotherapy.
2	Disconnect between the national and health facility-level perceptions on readiness to implement TIBA.	Misalignment in readiness at different levels can lead to implementation bottlenecks.	Establish regular cross-level communication and monitoring to ensure strategic alignment.
3	Leadership engagement	Strong leadership support facilitates implementation through motivation and resource mobilization.	Identify point persons/champions at all health systems levels to drive implementation
4.	Structural and resource limitations, workforce gaps, and procurement delays.	Lack of trained staff, essential commodities, and adequate space is a percussor to TIBA for implementation failure.	Targeted investments in capacity building, staff retention, and essential commodities. Streamline procurement services for cervical cancer screening services.
5.	Client recruitment challenges	High out-of-pocket costs reduce service uptake among clients in faith-based/ private health facilities	Promote cross-referrals and service integration between faith-based and public health facilities.
6.	Siloes and programmatic misalignment between implementing partners and the MOH	Misaligned priorities can lead to inefficient use of resources and demotivation among staff	Promote coordination between implementing partners and the MOH to integrate activities that align with implementing partners.

Our study has two limitations. First, we did not consistently obtain relevant provider participant characteristics, such as years of experience with cervical cancer preventive services and familiarity with the use of thermal ablation for same-day treatment. The lack of this detailed participant data may limit our ability to fully understand how these factors influenced the perspectives shared, thus potentially limiting the interpretation of our findings. Secondly, the questions assessing constructs related to the intervention characteristics domain focused primarily on comparing thermal ablation with other treatment modalities rather than evaluating the comprehensive single-visit screen-and-treat approach with thermal ablation (SV-SAT + TA). As a result, insights into the intervention characteristics domain's constructs may not fully capture perceptions of the overall strategy.

We obtained data from multiple stakeholders, including policymakers, health facility managers, and frontline providers offering reproductive health services across various health facility levels, providing a comprehensive understanding of the implementation context. This strengthens our findings’ robustness and applicability to policy and practice, making them more transferable to the Kenyan health context and other LMICs.

## Conclusion

This study aimed to identify implementation determinants of TIBA for cervical cancer prevention before the implementation trial, aiming to increase adoption. Participants highlighted key factors such as leadership engagement and the relative advantages of thermal ablation within the single visit, screen, and treat approach. Notably, the lower start-up and maintenance costs and thermal ablation's lower complexity than cryotherapy emerged as critical benefits. However, a lack of essential commodities for screening and treatment constrained human resources, and logistical and supply challenges emerged as drivers of implementation failure. Reducing silos within the healthcare system and fostering synergy between different health facility levels and management structures is crucial for strengthening the health system. A coordinated, integrated, system-wide approach involving all stakeholders is critical to ensuring the successful implementation of TIBA.

## Supporting information

S1 Table CFIR constructs. (DOCX)

S2 Table Thematic table. (DOCX)

S1 Text TIBA study protocol. (DOCX)

S1 Checklist Consort checklist. (DOC)

S2 Checklist Inclusivity. (DOCX)

## References

[1] Adams RA, Botha MH. Cervical cancer prevention in Southern Africa: A review of national cervical cancer screening guidelines in the Southern African development community. J Cancer Policy. 2024;40:100477. doi: 10.1016/j.jcpo.2024.100477 38593950

[2] Cancer Today. n.d. [cited 2024 Aug 19]. https://gco.iarc.who.int/today/

[3] IARC to host meeting of Expert Working Group on cervical cancer to review scientific evidence on screening programmes. n.d. [cited 2024 August 15]. https://www.iarc.who.int/cancer-type/cervical-cancer

[4] Cervical cancer. n.d. [cited 2024 November 24]. https://www.who.int/news-room/fact-sheets/detail/cervical-cancer

[5] Kawuki J, Savi V, Betunga B, Gopang M, Isangula KG, Nuwabaine L. Barriers to breast and cervical cancer screening among adolescent girls and young women in Kenya: A nationwide cross-sectional survey. Social Science & Medicine. 2025;367:117722. 39889379 10.1016/j.socscimed.2025.117722

[6] Cervical cancer causes, risk factors, and prevention. NCI. 2022 [cited 2024 September 2]. https://www.cancer.gov/types/cervical/causes-risk-prevention

[7] Cervical Cancer Elimination Initiative. World Health Organization. n.d. [cited 2024 September 2]. https://www.who.int/initiatives/cervical-cancer-elimination-initiative

[8] Anaba EA, Alor SK, Badzi CD, Mbuwir CB, Muki B, Afaya A. Drivers of breast cancer and cervical cancer screening among women of reproductive age: insights from the Ghana Demographic and Health Survey. BMC Cancer. 2024;24(1):920. doi: 10.1186/s12885-024-12697-6 39080553 PMC11290011

[9] Santesso N, Mustafa RA, Schünemann HJ, Arbyn M, Blumenthal PD, Cain J, et al. World Health Organization Guidelines for treatment of cervical intraepithelial neoplasia 2-3 and screen-and-treat strategies to prevent cervical cancer. Int J Gynaecol Obstet. 2016;132(3):252–8. doi: 10.1016/j.ijgo.2015.07.038 26868062

[10] WHO guideline for screening and treatment of cervical precancer lesions for cervical cancer prevention. n.d. [cited 2024 November 24]. https://www.who.int/publications/i/item/9789240030824

[11] Basu P, Meheus F, Chami Y, Hariprasad R, Zhao F, Sankaranarayanan R. Management algorithms for cervical cancer screening and precancer treatment for resource-limited settings. Int J Gynaecol Obstet. 2017;138 Suppl 1:26–32. doi: 10.1002/ijgo.12183 28691336

[12] Shin MB, Oluoch LM, Barnabas RV, Baynes C, Fridah H, Heitner J. Implementation and scale-up of a single-visit, screen-and-treat approach with thermal ablation for sustainable cervical cancer prevention services: a protocol for a stepped-wedge cluster randomized trial in Kenya. Implementation Science. 2023;18(1):26. 37365575 10.1186/s13012-023-01282-3 PMC10294443

[13] Interventions IWG on the E of CP. Screen-and-treat approach and women at differential risk. Cervical Cancer Screening. International Agency for Research on Cancer. 2022.

[14] Gitonga E, Iseme R, Mutisya R, Kodhiambo M. Cervical cancer knowledge, awareness and related health behaviours amongst women of reproductive age in Kiambu County, Kenya: a cross-sectional study. Health Psychol Behav Med. 2022;10(1):1056–70. 36299770 10.1080/21642850.2022.2136184 PMC9590427

[15] Amir SM, Idris IB, Said ZM, Yusoff HM, Manaf MRA. A Comparison of the National Cervical Cancer Policies in Six Developing Countries with the World Health Organization Recommendations: A Narrative Review. Iran J Public Health. 2023;52(6):1108–20. doi: 10.18502/ijph.v52i6.12952 37484154 PMC10362820

[16] Ng’ang’a A, Nyangasi M, Nkonge NG, Gathitu E, Kibachio J, Gichangi P. Predictors of cervical cancer screening among Kenyan women: results of a nested case-control study in a nationally representative survey. BMC Public Health. 2018;18(Suppl 3):1221. doi: 10.1186/s12889-018-6100-5 30400916 PMC6219012

[17] Mabachi NM, Wexler C, Acharya H, Maloba M, Oyowe K, Goggin K, et al. Piloting a systems level intervention to improve cervical cancer screening, treatment and follow up in Kenya. Front Med (Lausanne). 2022;9:930462. doi: 10.3389/fmed.2022.930462 36186820 PMC9520305

[18] Gebreegziabher ZA, Semagn BE, Kifelew Y, Abebaw WA, Tilahun WM. Cervical cancer screening and its associated factors among women of reproductive age in Kenya: further analysis of Kenyan demographic and health survey 2022. BMC Public Health. 2024;24(1):741. doi: 10.1186/s12889-024-18148-y 38459446 PMC10921781

[19] Kakotkin VV, Semina EV, Zadorkina TG, Agapov MA. Prevention Strategies and Early Diagnosis of Cervical Cancer: Current State and Prospects. Diagnostics (Basel). 2023;13(4):610. doi: 10.3390/diagnostics13040610 36832098 PMC9955852

[20] Li X, Chen S, Hirose N, Shimpuku Y. Association between multiple dimensions of access to care and cervical cancer screening among Kenyan women: a cross-sectional analysis of the Demographic Health Survey. BMC Health Serv Res. 2024;24:731. 38877555 10.1186/s12913-024-11169-8 PMC11177386

[21] Mwenda V, Murage D, Kilonzo C, Bor J-P, Njiri P, Osiro L, et al. Baseline assessment of cervical cancer screening and treatment capacity in 25 counties in Kenya, 2022. Front Oncol. 2024;14:1371529. doi: 10.3389/fonc.2024.1371529 39015502 PMC11249718

[22] Page CM, Ibrahim S, Park LP, Huchko MJ. Systems-level barriers to treatment in a cervical cancer prevention program in Kenya: Several observational studies. PLoS One. 2020;15(7):e0235264. doi: 10.1371/journal.pone.0235264 32658921 PMC7357749

[23] Petersen Z, Jaca A, Ginindza TG, Maseko G, Takatshana S, Ndlovu P. Barriers to uptake of cervical cancer screening services in low-and-middle-income countries: a systematic review. BMC Women’s Health. 2022;22:486. 36461001 10.1186/s12905-022-02043-y PMC9716693

[24] Diop A, Mvundura M, Dieng Y, Anne M, Vodicka E. Cervical cancer screening and treatment costing in Senegal. Pan Afr Med J. 2024;47:151. doi: 10.11604/pamj.2024.47.151.41485 38974700 PMC11226758

[25] Fact sheet - Latest global and regional statistics on the status of the AIDS epidemic. n.d. [cited 2024 March 18]. https://www.unaids.org/en/resources/documents/2023/UNAIDS_FactSheet

[26] World Health Organization. WHO guidelines for the use of thermal ablation for cervical precancer lesions. Geneva: World Health Organization. 2019. https://iris.who.int/handle/10665/329299

[27] National-Cancer-Screening-Guidelines-2018 copy.pdf.

[28] Pinder LF, Parham GP, Basu P, Muwonge R, Lucas E, Nyambe N, et al. Thermal ablation versus cryotherapy or loop excision to treat women positive for cervical precancer on visual inspection with acetic acid test: pilot phase of a randomised controlled trial. Lancet Oncol. 2020;21(1):175–84. doi: 10.1016/S1470-2045(19)30635-7 31734069 PMC6946855

[29] Zotero.pdf.

[30] Categorization of health institutions. 2023.

[31] Faith-Based Organizations. CapacityPlus. n.d. [cited 2024 December 3]. https://www.capacityplus.org/category/blog-tags/faith-based-organizations.html

[32] Means AR, Kemp CG, Gwayi-Chore M-C, Gimbel S, Soi C, Sherr K, et al. Evaluating and optimizing the consolidated framework for implementation research (CFIR) for use in low- and middle-income countries: a systematic review. Implement Sci. 2020;15(1):17. doi: 10.1186/s13012-020-0977-0 32164692 PMC7069199

[33] Crable EL, Biancarelli D, Walkey AJ, Drainoni M-L. Barriers and facilitators to implementing priority inpatient initiatives in the safety net setting. Implement Sci Commun. 2020;1:35. doi: 10.1186/s43058-020-00024-6 32885192 PMC7427845

[34] McEachern BM, Jackson J, Yungblut S, Tomasone JR. Barriers and facilitators to implementing exercise is medicine Canada on campus groups. Health Promotion Practice. 2019;20(5):751–9. doi: 10.1177/1524839918780775 30786774

[35] Rahimi S, Khatooni M. Saturation in qualitative research: An evolutionary concept analysis. Int J Nurs Stud Adv. 2024;6:100174. doi: 10.1016/j.ijnsa.2024.100174 38746797 PMC11080421

[36] Hennink M, Kaiser BN. Sample sizes for saturation in qualitative research: A systematic review of empirical tests. Social Science & Medicine. 2022;292:114523. 34785096 10.1016/j.socscimed.2021.114523

[37] Evaluation design – The Consolidated Framework for Implementation Research. n.d. [cited 2024 July 8]. https://cfirguide.org/evaluation-design/

[38] Home | Dedoose. n.d. [cited 2023 Oct 14]. https://www.dedoose.com/

[39] Mantula F, Toefy Y, Sewram V. Barriers to cervical cancer screening in Africa: a systematic review. BMC Public Health. 2024;24(1):525. doi: 10.1186/s12889-024-17842-1 38378542 PMC10877795

[40] Rauf L, Eid A, Hamed E. A global perspective on cervical cancer screening: a literature review. Int J Community Med Public Health. 2023;10(5):1942–6. doi: 10.18203/2394-6040.ijcmph20231044

[41] Moucheraud C, Kawale P, Kafwafwa S, Bastani R, Hoffman RM. Health care workers’ experiences with implementation of “screen and treat” for cervical cancer prevention in Malawi: A qualitative study. Implement Sci Commun. 2020;1(1):112. doi: 10.1186/s43058-020-00097-3 33317633 PMC7734769

[42] World Health Organization. Monitoring the building blocks of health systems: a handbook of indicators and their measurement strategies. Geneva: World Health Organization. 2010. https://iris.who.int/handle/10665/258734

[43] Rusmini R, Sukmawati E, Ruyani I, Albukhari Alidrus S, Muhammad Yusuf S, Samsu S. Building Strong Leadership through Individual Concept in Leading Pesantren. JMPIS. 2022;4(1):562–70. doi: 10.38035/jmpis.v4i1.1514

[44] Utilising VSM insights to address health system disconnects: introducing three novel organisational pathologies. University of Otago. n.d. [cited 2024 November 29]. https://ourarchive.otago.ac.nz/esploro/outputs/journalArticle/Utilising-VSM-insights-to-address-health/9926555799501891

[45] Negero MG, Sibbritt D, Dawson A. How can human resources for health interventions contribute to sexual, reproductive, maternal, and newborn healthcare quality across the continuum in low- and lower-middle-income countries? A systematic review. Hum Resour Health. 2021;19:54. 33882968 10.1186/s12960-021-00601-3 PMC8061056

[46] Kabene SM, Orchard C, Howard JM, Soriano MA, Leduc R. The importance of human resources management in health care: a global context. Hum Resour Health. 2006;4:20. doi: 10.1186/1478-4491-4-20 16872531 PMC1552082

[47] Shikuku DN, Nyaoke I, Maina O, Eyinda M, Gichuru S, Nyaga L. The determinants of staff retention after Emergency Obstetrics and Newborn Care training in Kenya: a cross-sectional study. BMC Health Services Research. 2022;22:872. 35794569 10.1186/s12913-022-08253-2 PMC9261014

[48] Walters C, Cope V, Hopkins MPR. Left behind: Exploring the concerns of emergency department staff when personnel are utilised for inter-hospital transfer. Int Emerg Nurs. 2023;69:101298. doi: 10.1016/j.ienj.2023.101298 37257361

[49] Arnold SA, Meyer NL, Tonaus S, Shaffer BL, Baniak LM. Implementation and Evaluation of a Nurse Practitioner Onboarding Program at a Large Healthcare Facility. J Nurs Adm. 2023;53(10):515–9. doi: 10.1097/NNA.0000000000001327 37747174

[50] Agarwal S, Glenton C, Henschke N, Tamrat T, Bergman H, Fønhus MS, et al. Tracking health commodity inventory and notifying stock levels via mobile devices: a mixed methods systematic review. Cochrane Database Syst Rev. 2020;10(10):CD012907. doi: 10.1002/14651858.CD012907.pub2 33539585 PMC8094928

[51] Martineau T, Ozano K, Raven J, Mansour W, Bay F, Nkhoma D, et al. Improving health workforce governance: the role of multi-stakeholder coordination mechanisms and human resources for health units in ministries of health. Hum Resour Health. 2022;20(1):47. doi: 10.1186/s12960-022-00742-z 35619105 PMC9134719

[52] Niyonsenga G, Gishoma D, Sego R, Uwayezu MG, Nikuze B, Fitch M, et al. Knowledge, utilization and barriers of cervical cancer screening among women attending selected district hospitals in Kigali - Rwanda. Can Oncol Nurs J. 2021;31(3):266–74. doi: 10.5737/23688076313266274 34395829 PMC8320790

[53] Patient satisfaction in FBO.pdf.

[54] Evaluation of Health Care-Nyongesa.pdf.

[55] Shumba CS, Kabali K, Miyonga J, Mugadu J, Lakidi L, Kerchan P, et al. Client satisfaction in a faith-based health network: findings from a survey in Uganda. Afr Health Sci. 2017;17(3):942–53. doi: 10.4314/ahs.v17i3.38 29085423 PMC5656196

[56] Binyaruka P, Anselmi L. Understanding efficiency and the effect of pay-for-performance across health facilities in Tanzania. BMJ Glob Health. 2020;5(5):e002326. doi: 10.1136/bmjgh-2020-002326 32474421 PMC7264634

[57] Chakraborty NM, Montagu D, Wanderi J, Oduor C. Who serves the poor? An equity analysis of public and private providers of family planning and child health services in Kenya. Front Public Health. 2019;6:374. 30671427 10.3389/fpubh.2018.00374 PMC6331399

[58] Mercer T, Gardner A, Andama B, Chesoli C, Christoffersen-Deb A, Dick J, et al. Leveraging the power of partnerships: spreading the vision for a population health care delivery model in western Kenya. Global Health. 2018;14(1):44. doi: 10.1186/s12992-018-0366-5 29739421 PMC5941561

[59] Lau RS, Boesen ME, Richer L, Hill MD. Siloed mentality, health system suboptimization and the healthcare symphony: a Canadian perspective. Health Res Policy Syst. 2024;22:87. 39020412 10.1186/s12961-024-01168-w PMC11253392

[60] Brown H. Global health partnerships, governance, and sovereign responsibility in western Kenya. American ethnologist. 2015;42(2):340–55.

[61] Barr A, Garrett L, Marten R, Kadandale S. Health sector fragmentation: three examples from Sierra Leone. Global Health. 2019;15(1):8. doi: 10.1186/s12992-018-0447-5 30670026 PMC6341573

