# Supplementary material for: Barriers and facilitators to implementing a single-visit, screen-and-treat approach with thermal ablation for cervical cancer prevention in Kenya
Source: PLOS Glob Public Health. 2025 Sep 9;5(9):e0005166. doi: 10.1371/journal.pgph.0005166 (PMC12419645; doi:10.1371/journal.pgph.0005166)
Supplement: S1 Table — (DOCX) [file pgph.0005166.s001.docx]

**S1 Table**. CFIR Constructs included in the question guide

| **Domain** | **Constructs** | **Sample Questions** |
| --- | --- | --- |
| Intervention Characteristics | Relative advantage | What demonstrates that TIBA is better or worse than existing programs? |
|  | Adaptability | What kind of alterations will be needed to make TIBA work effectively? |
|  | Complexity | How complicated is thermal ablation? |
|  | Evidence strength and quality | What kind of evidence are you aware of that  shows whether or not TIBA will work in your setting? |
|  | | |
| **Inner Setting** | Structural Characteristics | How will the infrastructure of your organization (social architecture, age, maturity, size or physical layout) affect TIBA implementation  What infrastructural changes will be needed to  accommodate TIBA? |
|  | Networks and communication | What is the general communication and  relationships in the organization? |
|  | Readiness for implementation Leadership engagement  Available resources | What level of support have you received or heard from your leaders to make implementation successful  Do you expect to have sufficient resources to implement TIBA? |
|  | Implementation climate | What is the general level of receptivity in your  organization to implementing SVA-SAT+TA? |
|  | | |
| **Characteristics of Individuals** | Self-efficacy | How confident are you that you will be able to deliver TIBA? |
|  | Individual stage of change | How prepared are you to implement TIBA? |
|  | Knowledge and beliefs about innovation | What do you know about TIBA, and do you think it will be effective in your setting? |
|  | | |
| **Characteristics of**  **Systems** | System architecture/health  system context | What health system factors may affect TIBA  implementation? |
|  | Policies, programs and  guidelines | What policies, programs, and guidelines may  affect TIBA implementation? |
|  | | |
| **Outer Setting** | Patient needs and resources | How well do you think TIBA will meet the needs of women screening for cervical cancer and  treatment in your facility? |
