# Supplementary material for: Barriers and facilitators to implementing a single-visit, screen-and-treat approach with thermal ablation for cervical cancer prevention in Kenya
Source: PLOS Glob Public Health. 2025 Sep 9;5(9):e0005166. doi: 10.1371/journal.pgph.0005166 (PMC12419645; doi:10.1371/journal.pgph.0005166)
Supplement: S2 Table — (DOCX) [file pgph.0005166.s002.docx]

**S2 Table.** Thematic Table

| **Domain/Themes** | **Constructs/Codes** |  | **Illustrative Quotes** |
| --- | --- | --- | --- |
| **Innovation Characteristics** | **Relative advantage and complexity** | What demonstrates that TIBA is better or worse than existing programs?  How complicated is thermal ablation? | *It is easier and less complex compared to cryotherapy. It does not require regular filling of the consumables. It's portable and can potentially improve linkage to care (Policymaker, 003).* |
|  | Adaptability | What kind of alterations will be needed to make TIBA work effectively? |  |
|  | Evidence strength and quality | What kind of evidence are you aware of that shows whether or not TIBA will work in your setting? |  |
| **Inner Setting** |  |  |  |
|  | **Structural Characteristics** | How will the infrastructure of your organization (social architecture, age, maturity, size or physical layout) affect TIBA implementation  What infrastructural changes will be needed to accommodate TIBA? | *Our rooms are tiny, the ones the partners had the structure for the CCC (HIV treatment rooms. (Clinical Officer, 009)* |
|  | Networks and communication | What is the general communication and relationships in the organization? |  |
|  | Readiness for implementation  **Leadership engagement**  **Available resources** | What level of support have you received or heard from your leaders to make implementation successful  Do you expect to have sufficient resources to implement TIBA? | *I think they [policymakers] are well versed that they can advise and guide on developing appropriate guidelines. The policies are already there for screening, and there is relevant expertise to be able to guide that process (Policymaker, 004)*  *We need refresher training, the actual machine (TA), supportive supervision, and mentorship from the national government. If we can get staff employed, this will address the staff shortage (Nurse, 004).* |
|  | Implementation climate | What is the general level of receptivity in your organization to implementing SVA-SAT+TA? |  |
| **Characteristics of Individuals** |  |  |  |
|  | **Self-efficacy** | How confident are you that you will be able to deliver TIBA? | *I would not say I am very confident. You know it is a new thing. So, we are learning and eager to learn, but I cannot predict how the patients and health workers will take it, so I am eagerly waiting. (Obstetrician-Gynecologist 010)* |
|  | Individual stage of change | How prepared are you to implement TIBA? |  |
|  | Knowledge and beliefs about innovation | What do you know about TIBA, and do you think it will be effective in your setting? |  |
| **Characteristics of Systems** |  |  |  |
|  | **System architecture/health system context** | What health system factors may affect TIBA implementation? | *So, the procurement, I said, is a long process. When our gas [for cryotherapy] went off, we stayed for six months, and one of the partners bought for us that gas, the one that we are using currently. (Nurse, 007)*  *Implementing population-based screening may be challenging because, despite having screening guidelines, there is no system to track which patients complete screening, follow up on results, or return for care. (Policymaker, 004).* |
|  | **Policies, programs and guidelines** | What policies, programs, and guidelines may affect TIBA implementation? |  |
| **Outer Setting** |  |  |  |
|  | Patient needs and resources | How well do you think TIBA will meet the needs of women screening for cervical cancer and treatment in your facility? |  |

*Bold constructs: Most important constructs frequently mentioned as influencing factors to TIBA implementation.*
