# Supplementary material for: Barriers and facilitators to implementing a single-visit, screen-and-treat approach with thermal ablation for cervical cancer prevention in Kenya
Source: PLOS Glob Public Health. 2025 Sep 9;5(9):e0005166. doi: 10.1371/journal.pgph.0005166 (PMC12419645; doi:10.1371/journal.pgph.0005166)
Supplement: S1 Text — (DOCX) [file pgph.0005166.s003.docx]

**PROTOCOL AND IMPLEMENTATION PLAN**

**Towards cervical cancer elimination: Implementation and scale-up of a** **single-visit, screen-and-treat approach with thermal ablation for sustainable cervical cancer prevention services in Kenya**

***TIBA STUDY-Towards a cervical cancer free generation***

**Version 3.0**

**13th November 2023**

***Funding:***

***United States National Institute of Health (NIH)***

**IMPLEMENTATION LEADERSHIP TEAM**

***University of Washington, Seattle , USA***

Sarah Gimbel, MPH, PhD

Leeya Pinder, MD, MPH

Renee Heffron, MPH, PhD

Ruanne Barnabas MBChB, DPhil

Deborah Donnell, PhD

Darcy Rao, PhD

***Kenya Medical Research Institute, Nairobi, Kenya***

Nelly Rwamba Mugo, MBChB, MMed Obs/Gyn, MPH

Lynda Oluoch, MBChB, MSc, MPH

***Jomo Kenyatta University of Agriculture and Technology, Nairobi, Kenya***

Kenneth K. Ngure, MPH, MSc, PhD

***Kisumu Medical and Educational Trust***

Monica Oguttu, PhD

**TABLE OF CONTENTS**

**Contents**

[ABBREVIATIONS 4](#_Toc93526670)

[EXECUTIVE SUMMARY 5](#_Toc93526671)

[BACKGROUND AND RATIONALE 7](#_Toc93526672)

[INNOVATION 14](#_Toc93526673)

[STUDY OBJECTIVES 15](#_Toc93526674)

[STUDY DESIGN 15](#_Toc93526675)

[IMPLEMENTATION PLAN: 16](#_Toc93526676)

[LAB PROCEDURES 29](#_Toc93526677)

[DATA 29](#_Toc93526678)

[STUDY LIMITATIONS 31](#_Toc93526679)

[HUMAN SUBJECTS CONSIDERATIONS 32](#_Toc93526680)

[EXPECTED APPLICATION OF RESULTS 36](#_Toc93526681)

[STUDY TIMELINE 36](#_Toc93526682)

[REFERENCES 37](#_Toc93526683)

# **ABBREVIATIONS**

CC Cervical cancer

SVA Single Visit Approach

SAT Screen and Treat

LMIC Low and middle income countries

WHO World Health Organization

TA Thermal ablation

D&I Dissemination and Implementation

RE-AIM Reach, Effectiveness, Adoption, Implementation and Maintenance

RH Reproductive Health

NACC National Control Cancer Program

IDI In-depth Interviews

CFIR Consolidated Framework for Implementation Research

HPV Human Papilloma Virus

DHIS Demographic Health Information System

KNH Kenyatta National Hospital

LEEP Loop Electrosurgical Excision Procedure

VIA Visual Inspection with Acetic acid

VILI Visual Inspection with Lugol’s Iodine

IRB Institutional Review Board

KEMRI Kenya Medical Research Institute

CIN Cervical Intraepithelial Neoplasia

NIH National Institutes of Health (US)

HIV Human Immunodeficiency Virus

ORIC Organizational Readiness for Implementing Change

STI Sexually transmitted infection

PrEP Pre-exposure prophylaxis

IS Implementation Science

KMET Kisumu Medical and Educational Trust

US United States

UW University of Washington

VCT Voluntary counseling and testing

MoH Ministry of Health

PHRD Partners in Health and Research Development

KEMRI Kenya Medical Research Institute

ICRC International Clinical Research Centre

CME Continuous Medical Education

QA Quality Assurance

UoN University of Nairobi

TDABC Time-driven activity-based costing

# **EXECUTIVE SUMMARY**

Cervical cancer (CC) is almost entirely preventable with current technologies, yet, it remains the 4^th^ most common cause of cancer incidence and mortality among women globally.(1) CC is the most common cause of cancer and cancer deaths among women in Kenya, and 90% of CC deaths occur in low- and middle- income countries (LMIC).(2) To achieve the 2018 World Health Organization (WHO) call to action towards global CC elimination, there is an urgent need to adapt, implement, and scale-up effective technologies in LMICs.(3, 4)

The mainstay of CC prevention in LMIC has been the single-visit approach with screen-and-treat (SVA-SAT) method, using visual inspection with acetic acid (VIA) and ablative treatment with cryotherapy to manage precancerous lesions. It is a low-cost screening approach and minimizes loss to follow-up compared to traditional cytology.(5) Despite well-established evidence of the effectiveness of VIA on population-level reduction in CC burden,(6-8) the estimated screening uptake among women aged 30-49 in Kenya is 16%,(9) far below the WHO’s target of 70% and twice-lifetime screening of women ages 35-45 by 2030.(3) In Kenya, there is extremely low fidelity of SVA-SAT; up to 70% of screen-positive women do not receive treatment, demonstrating the science-practice gap.(10) The low treatment rate has been attributed to programmatic and logistical challenges of implementing cryotherapy in low-resource settings (e.g., equipment shortage, costs, supply chain difficulties of refrigerant gas, equipment failure, and treatment duration >10 min).(11-13) Thermal ablation (TA) is an effective alternative to cryotherapy for ablation of precancerous lesions, and has been recommended by the WHO since 2019.(14) Preliminary data from an investigative team member demonstrated safety, effectiveness, and acceptability to women when delivered by nurses in Zambia and Kenya.(15, 16) The portable device can be charged with electricity, batteries or solar panels, which is ideal for low-resource settings.(17) Successful implementation and scale-up of TA within the SVA-SAT approach could optimize CC prevention.

Testing dissemination and implementation (D&I) models to achieve population-based CC prevention is a top priority in Kenya’s National Cancer Control Strategy.(18) A context-informed scale-up of the SVA-SAT+TA in reproductive health (RH) clinics will be essential to achieve this goal. We have assembled a multidisciplinary team, including the head of the Division of National Control Cancer Program (NCCP) and TA experts, to advance the pragmatic delivery of SVA-SAT+TA. We propose a five-year prospective, stepped-wedge, cluster randomized trial to implement SVA-SAT+TA in 10 reproductive health (RH) clinics in central Kenya. We will use mixed-methods evaluation based on the RE-AIM (Reach, Effectiveness, Adoption, Implementation and Maintenance) framework to assess the intervention’s impact. We will collaborate with multi-level (clinic, county, national) stakeholders to develop a sustainable D&I strategy and leverage trial implementation to include costing and budget impact analysis. Our objective is to develop and evaluate a locally contextualized dissemination and implementation (D&I) strategy for SVA-SAT with TA (SVA-SAT+TA) to inform national scale-up. Our hypothesis is that TA will enhance the feasibility, adoption, and sustainability of CC prevention services via SVA-SAT, compared to the standard of care with cryotherapy.

**Design**:

Prospective, stepped-wedge, cluster randomized trial to implement SVA-SAT+TA in ten reproductive health (RH) clinics in central Kenya.

**Population**: Mixed population: Reproductive health care workers, health manager’s, women seeking services for cervical cancer screening and women who screen positive for pre-cancerous lesions of the cervix.

**Objective 1:**

**Develop a dissemination and implementation strategy to introduce SVA-SAT+TA that effectively accounts for the heterogeneity of the client, provider, and system inputs**.

*Approach*: Building upon our experiences with TA in Zambia,(13) we will conduct in-depth interviews (IDIs) with clients (n=20), providers (n=20), managers (n=10), and NCCP stakeholders (n=5) to identify actionable barriers and facilitators to SVA-SAT+TA uptake. Using a participatory feedback approach, we will conduct a stakeholder workshop to synthesize and contextualize strategies to effectively introduce the intervention and provide optimized implementation guidance for scaling.

**Objective 2:**

**Deliver the SVA-SAT+TA intervention at scale in RH clinics and evaluate implementation using the RE-AIM framework**.

*Approach*: We will introduce the SVA-SAT+TA into RH clinics using a stepped-wedge study design and rigorously evaluate how effectively this intervention is disseminated and implemented. Key endpoints will include:

a) (REACH) Proportion of the clinics reached, providers trained;

b) (EFFECTIVENESS) Intervention effect on SVA-SAT process measures: number of screen positive women identified per month, treatment completion rate compared to pre-intervention, and the patient-level TA fidelity of implementation assessed by testing HPV clearance 6 months post-treatment among 360 randomly selected screen positive women treated with TA;

c) (ADOPTION) Proportion of the clinics incorporating TA into routine care through updated policies and trained provider in practice;

d) (IMPLEMENTATION) Determine core components of the SVA-SAT+TA intervention and describe drivers of success/failure using the Consolidated Framework for Implementation Research;

e) (MAINTENANCE) Proportion of clinics that continue to provide and sustain SVA-SAT+TA services 6-12 months after intervention implementation.

**Objective 3:**

**Compare the cost and budget impact of SVA-SAT+TA to SVA-SAT using cryotherapy.**

*Approach*: We will use micro-costing techniques to quantify the programmatic costs of SVA-SAT with cryotherapy and with TA. The strategies will be compared in terms of the cost per woman treated from the programmatic perspective, with a secondary analysis incorporating costs incurred by patients. A budget impact analysis will account for the size of the patient population, costs averted and opportunity costs from the Ministry of Health perspective.

# **BACKGROUND AND RATIONALE**

**Importance of the problem**

**Global elimination of cervical cancer is a feasible goal**; however, the countries with the greatest disease burden also have the greatest healthcare system challenges. Cervical cancer (CC) is almost entirely preventable. It is one of the few cancers with highly effective vaccines for primary prevention (the human papillomavirus (HPV) vaccine), and low-cost evidence-based technologies for effective early detection and treatment as secondary prevention. Yet CC ranks as the 4^th^ most common cancer and cause of cancer deaths among women globally, each year 569,847 women are diagnosed with invasive CC, and 311,000 die from this preventable cancer.(2) Most morbidity and mortality associated with CC occur in women in sub-Saharan Africa (Fig.1), highest in East and southern Africa, and women living in low- and middle-income countries (LMICs) bear 90% of the CC mortality. CC is especially destabilizing, as it affects women in their reproductive years, when they are caregivers and economic providers for their families.

**Figure 1: Age standardized (World) incidence and mortality rates, cervix uteri**


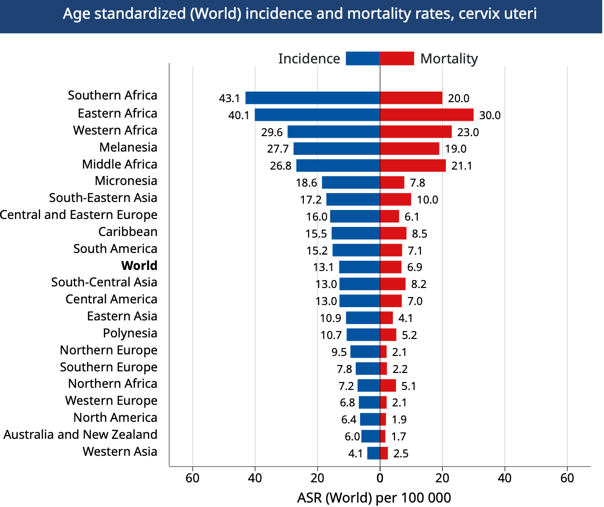


*Source: Global Cancer Observatory (Cervix uteri* [*(https://gco.iarc.fr/today/data/factsheets/cancers/23-Cervix-uteri-fact-sheet.pdf)*](https://gco.iarc.fr/today/data/factsheets/cancers/23-Cervix-uteri-fact-sheet.pdf)

The global disproportionate burden of CC placed on low resource settings, is evidenced by the wide range in age-standardized incidence estimates (Fig. 1), which range from 2 to 75 per 100,000 women.(2, 19) High-income countries had equally high rates of CC morbidity and mortality prior to the implementation of screening programs with high population coverage. The United Kingdom, as an example, in 1988, increased screening coverage through a national screening program and reduced CC incidence within a decade by 35%.(20) The current low incidence rates in high resource settings reflect the effectiveness of proven interventions and the contrasting high incidence rates in low resource setting reflect inequity in access to preventive services, including CC screening services and the HPV vaccine. In Kenya, CC is the most common cancer and most common cause of cancer-related death among women, with an age standardized incidence of 33.8/100,000 and 5250 new CC cases and 3286 deaths annually.(21) In May 2018, the World Health Organization (WHO) Director General made a global call for action towards the elimination of CC.(22) **To achieve this critical goal quickly and equitably, the investment must be made to effectively adapt, deliver, and scale-up proven screening and treatment methods in low resource settings.**

**Access to secondary prevention interventions is critical to achieving cervical cancer elimination**. In the next 50 years, no single intervention will eliminate CC. According to the WHO CC elimination modelling consortium, the impact of reaching the WHO elimination target of 70% coverage of women 35-45 years screened and 90% CC treated, combined with 90% HPV vaccination coverage for girls <15 years (WHO’s triple intervention 90-70-90 strategy) would reduce 30% of CC deaths, averting approximately 300,000 deaths by 2030, and 92% by 2070.(23, 24) The impact of HPV vaccination alone on CC in a decade would be <1%, and is anticipated to increase to 62% by 2070, reducing the proportion of deaths averted with secondary prevention and achieving near elimination by 2120. The modeling consortium anticipates that 50% of the CC deaths averted within the next decade with implementation of the WHO’s triple intervention strategy would occur in sub-Sahara Africa. If we retain status quo, 50% increase in CC deaths is expected.

**In Kenya, national cervical cancer prevention screening and treatment rates are low.** Secondary prevention of CC will effectively reduce disease burden, only when simultaneous diagnosis and linked treatment of precancerous lesions is prioritized. Effectiveness of treatment interventions is largely dependent on the adaptability and flexibility of the CC diagnostic tools and their performance across differing environments with practical considerations that promote screening access for eligible women. Therefore, testing dissemination and implementation (D&I) models to achieve population-based CC prevention is a top priority in Kenya’s National Cancer Control Strategy for 2017-2022.(18) The 2015 Kenya STEPwise survey report on non-communicable diseases estimates that only 16.4% of women age 30-49 have been screened for cervical cancer,(9) and the Kenya 2019 demographic health information systems (DHIS-www.health.go.ke/password required) data further shows that only a third of screen positive women eventually access treatment.(10) These national screen and treatment rates fall far below the WHO’s 90-70-90 triple intervention strategy goals.(3) There were no reports of treatment with thermal ablation for treatment of precancerous CC lesion on the national STEPwise survey. At the Kenyatta National Hospital (KNH) colposcopy clinic in Kenya, we found a delay of 24 weeks between cytology (Pap test) diagnosis of high-grade lesions and treatment with excision using loop electrosurgical excision procedure (LEEP).(25) Our preliminary results confirms and underlines the challenge of implementing a cytology and excision-based program in low resource settings, even with access to skilled providers.

**Single-visit approach with screen-and-treat method for cervical cancer screening programs is preferable for LMIC**. CC has a long process of carcinogenic transformation from high risk human papillomavirus (hrHPV) infection to development of precancerous lesions and invasive cancer, providing ample opportunity to detect and treat, when treatment is effective. The effectiveness of visual inspection with acetic acid (VIA), HPV test and cervical cytology for early detection of precancerous lesions of the cervix, and the cost-effectiveness of screening programs has been well-established. (26, 27) The WHO 2016 guidelines endorsed screening with VIA using the single-visit approach to screen-and-treat (SVA-SAT) with cryotherapy for treatment of screen positive lesions, which has been the mainstay of CC prevention in LMICs.(5, 28) Compared to the traditional cytology-based screening, VIA is cheaper, less labor intensive, does not require specialized laboratory services and minimizes loss to follow-up. Women prefer the SVA-SAT method because it averts the anxiety of waiting for diagnosis and reduces the burden of multiple clinic visits.(29)

**Cryotherapy and thermal ablation are safe and effective treatment for precancerous lesions of the cervix.** Treatment of pre-invasive cervical lesions can be achieved through ablation or excision of the lesion. Ablative techniques using cryotherapy and thermal ablation destroy abnormal cervical cells and can be effectively and safely performed by nurses in primary care settings.(14, 27) Excisional procedures are effective and provide confirmatory diagnosis with tissue biopsy, but require expensive infrastructure, highly skilled practitioners, may impact fertility and have a higher risk of surgical complications. Cryotherapy has been in use for many years for treatment of precancerous cervical lesions with indisputable documentation of effectiveness (Table 1).(30) It was the recommended treatment option for LMICs by the WHO 2013 and 2016 cervical cancer screening guidelines.(28) Effectiveness has been demonstrated with consistently similar cure rates in different settings of 94% for CIN1, 92% for CIN2 and 85% for CIN3 and can be safely and effectively performed in primary health care settings.(31) Implementation, however, has a major logistic drawback, the large gas tanks of compressed refrigerant gas required to cool the probe used for treatment are difficult to procure and transport in LMICs primary care settings. It is also time-consuming, requiring 12 minutes for full treatment.

**Table 1: Evidence for Single Visit Approach with Screen and Treat**

| **Study** | **Country** | **Intervention** | **Provider** | **Cure rates** |
| --- | --- | --- | --- | --- |
| Sankaranarayanan. Brit J. Cancer 2007 | India | VIA/ Cryotherapy | Nurse | 81.4% CIN 1  70.6% CIN 2+ |
| Blumenthal P. Am J Obstet. 2007 | Ghana | VIA/Cryotherapy | Nurse | 97.4% |
| [B Chumworathayi](https://pubmed-ncbi-nlm-nih-gov.offcampus.lib.washington.edu/?term=Chumworathayi+B&cauthor_id=17949424)  et al. Int J Gynecol Cancer 2008 | Thailand | VIA/ Cryotherapy | Nurse | 85.5% |
| Denny L. JAMA 2005 | South Africa | VIA/Cryotherapy | Nurse | 94.6% |
| Chigbu C. Niger J Clin Pract. 2017 | Nigeria | VIA/ Cryotherapy | Nurse | 87.9% |
| Sauvaget C. Meta-analysis Int J. Gyn Obst. 2013 | HIC & LMIC | Cryotherapy | Mid-level | 92% CIN-2 85% CIN-3 |
| Dolman L. Meta-analysis. BJOG. 2014 | HIC | Thermal ablation | Mid-level | 96% CIN-1  95% CIN2+ |
| Randall TC. Meta-analysis. Preventive Med. 2018 | HIC & LMIC | Thermal ablation |  | 93.8% CIN 2+ |

HIC: high-income country

Thermal ablation has comparative advantage over cryotherapy (Table 2), it can be recharged with either electricity, solar-energy or even a car battery, treatment is shorter than cryotherapy (20-45 seconds per application), the instrument is small with portable options,(32) has minimal infrastructure requirements, probes are easy to sterilize and treatment can be completed by mid-level providers in primary care settings.(13) Thermal ablation has well documented outcomes, was commonly used in the Europe, especially the UK, for treatment of cervical precancerous lesions among other gynecological conditions in the 1980s. A metanalysis of thermal ablation by Dolman et al. in 2013, and an updated 2018 review by Randall et al., which included publications from LMICs, found cure rates of 94% for CIN2+ with treatment using thermal ablation (Table 1).(33)^,^(17) Reported side effects were mild to moderate and similar to cryotherapy, with vaginal discharge lasting 1-3 weeks, and mild pain post procedure and the majority reporting no side effects. Results from a more recent randomized clinical trial conducted in Zambia by a member of our investigative team(13) (Leeya Pinder) and colleagues, revealed similar cure rates among women treated with thermal ablation (64%), cryotherapy (60%) and in the LLETZ (67%), as evaluated by assessing clearance of HPV infection 6 months after treatment.(16) Complaints of pain were document in approximately 2% of patients across treatment groups and no pain in the thermal ablation group were noted 2 weeks after procedure. More recent data from Western Kenya, found TA acceptability at 99%(16). This cumulative evidence has led to the adaptation of thermal ablation as treatment for pre-cancer lesions of the cervix and inclusion of thermal ablation as standard of care as recommendation by the 2019 WHO guidelines.(14)

**Table 2: Comparing Cryotherapy and Thermal Ablation**

**Kenya National Cervical Cancer program (NCCP) is committed to cervical cancer prevention.** Kenya 2017-2022 national cancer control strategy has identified prevention, screening and early detection as the first of five strategic objectives to cancer control.(34) The 2018 cervical cancer screening guidelines propose to have every woman age 25-49 screened regularly for cervical cancer.(35) Despite the well-established evidence on the effectiveness of VIA on the population-level reduction in cervical cancer burden, the screening coverage and treatment of screen positive lesions remain low.(36) In an effort to expand screening coverage the Kenya NCCP has an on-going pilot project, which is field testing HPV testing for screening in six counties in Kenya. Concurrently, the NCCP is conducting a survey in public facilities to assess equipment coverage for cervical cancer control services. Over the last two decades various projects have introduced screening and treatment equipment to public health facilities, but the services often cease once the project ends. In Kenya, the gap that exists is between the decision to adopt an intervention (cervical cancer prevention policy and guidelines) and the routine use of interventions (VIA and cryotherapy). The lack of intervention uptake is reflected on inadequately skilled providers at RH clinics, inconsistent use of cryotherapy following screening and lack of community awareness.(37) The barriers and facilitators of this inadequate uptake require further interrogation to inform a sustainable program. Preliminary discussions with health care providers providing services at 12 potential project RH clinics in central Kenya, revealed challenges consistently related to cryotherapy equipment and procurement of supplies. Among clinics surveyed with cryotherapy equipment, challenges included equipment failure, high cost of refrigerant gas, and high turnover of trained personnel. They reflect challenges identified in published literature.(26)

**Preliminary data from the Zambia cervical cancer prevention program with the use of thermal ablation.** There are few LMICs that have been able to implement, sustain and scale-up a cervical cancer screen and treat program, especially in sub-Saharan Africa.(28) In Zambia, where cytology and HPV testing are not routinely available, trained nurses perform low-cost screening using WHO-recommended VIA enhanced with digital photography of the cervix (digital cervicography), using a commercial brand, off-the-shelf digital camera to screen women for cervical pre-cancer at the primary health care level.(38) Women with simple cervical lesions are treated at the primary health care level by nurses using ablation with cryotherapy and more recently, with years of country data to support its feasibility and acceptability, thermal coagulation is being introduced throughout the country (unpublished data). Those with complex cervical lesions are referred to secondary (district and provincial) or tertiary hospitals for histopathologic evaluation via punch biopsy or local outpatient excision of the abnormal cervical lesion using LEEP. Since inception, Zambia’s cervical cancer screening program has been scaled up to 60 of Zambia’s health clinics covering all 10 provinces and has provided screening and treatment services to over 600,000 women. For Zambia, VIA satisfies many of the prerequisites for an effective low-resource environment screening test, yet treatment using cryotherapy has limited consistency in which same-day treatment can be provided. Their challenges are similar to the experiences of other countries, expense of refrigerant gas required for treatment, fractured gas-supply chain and time per treatment cycle. With the use of thermal coagulation, these challenges are more easily overcome. *Reported by a team member (L. Pinder), who works with the Zambia’s cervical cancer screening program.*

**Implementation research is needed to develop strategies for context-tailored intervention delivery.** The investment and advances in development of highly effective interventions to diagnose and treat pre-cancerous lesions of the cervix has not borne the anticipated promise of protecting the 569,000 women diagnosed and 313,365 who die from CC each year.(2) This promise can only be achieved with scale-up of screening services with population level coverage and providing access to effective, appropriate management of screen-positive lesions for women. Implementation research is needed to address the evidence-to-practice gap in implementation of contextually appropriate cervical cancer prevention programs. Implementation of cervical cancer screening programs in low- and middle-income countries remains understudied. Dykens, et al. found only 5 of 51 implementation science published articles on cervical cancer to be from low income countries, despite their higher disease burden and weaker health systems.(39) Comparatively, prevention of mother to child HIV transmission programs, in similar resource limited settings, have benefited from a wealth of implementation science research, with substantial contributions from Dr. Gimbel, a member of our investigative team, bringing the world closer to the elimination goal.(40-42)

Contextually tailored implementation evidence is needed to guide effective scaling of thermal ablation and the single visit screen and treat approach in health facilities to bridge access to CC services for women in low resource settings and inform scaling for national programs. In this study we propose use of implementation science methods to understand and explain what influences implementation outcomes (including the Consolidated Framework for Implementation Research (CFIR) within a RE-AIM evaluative framework.(43) With CFIR, a determinants framework, we will explore implementation variability across clinics in order to discern and contextualize drivers of implementation success and failure. To understand readiness for adopting SVA-SAT+TA across implementing facilities we will apply the Organizational Readiness for Implementing Change (ORIC) assessment scale.(44) Many efficacious health interventions are not successfully institutionalized because they have been tested in controlled settings. Subsequently, when these interventions are applied to the general population, their effectiveness lessens, dampening enthusiasm for scaling efforts .(45) The RE-AIM framework , which will be used as the theoretical framework for this study, improves the applicability and generalizability of research findings by balancing internal and external validity during the development and testing of interventions,(46) through measurement of reach, effectiveness, adoption and implementation of SVA-SAT+TA. This implementation focused approach will harness the community of health system frontline workers and managers as partners in project implementation and build ownership of results, with an aim towards supporting maintenance after we stop research intervention activities.

**Foundation for proposed work**: Our team has vast experience in clinical management of pre-cancerous cervical lesions (47-52) including use of thermal ablation and training of health care providers.(13) At the KMET supported clinics, we have high CC screening rates, with more than 20,000 screened for CC (2018 – 2019) and high treatment completion rates.(53) Dr. Mugo in conjunction with the PAP training team has conducted training camps for training RH specialists in Kenya on colposcopy and LEEP at the Kenyatta National Hospital (2000) and the Embu Level 5 hospital, (2002) where those services continue to be provided. In addition, she has worked with public health clinics implementing stepped wedge trials in the Partners Scale Up Project. Our team is at the forefront of PrEP scale up in Kenya and we are experienced in conducting implementation science evaluations to catalyze scale up of new interventions through health provider training and provision of technical assistance (54) and use of participatory approaches to refine new interventions.(55)

**Justification for stepped wedge design.** Stepped wedge designs use sequential roll-out of an intervention (to individuals or clusters – in the present case, RH clinics) over time to assess the impact of an intervention, with the order of receiving the intervention determined at random.(56) The design is especially relevant for proven interventions that cannot be withheld ethically, but which cannot necessarily be moved to scale simultaneously in all the clinics with the resources at hand. All clusters are thus offered the intervention by the end of the study, with the order of initiation randomly assigned. Prior to a cluster offering the intervention, the cluster contributes data to the “pre-implementation” (e.g., control) period and once the cluster has begun to implement the intervention, it contributes to the “intervention” period. This design allows for comparisons of key outcomes in the intervention versus control periods. SVA-SAT+TA is an ideal intervention candidate for a cluster-randomized stepped wedge design, given that it is 1) an evidence-based intervention, 2) it is logistical and feasible to scale in a phased fashion (which then allows for assessment of implementation with each wave), and 3) is implemented at the unit of the facility (i.e., thus we can randomize to phase of implementation at the clinic level). This design also allows us to quantify the effect of the intervention on screening coverage and treatment effectiveness, while providing data on background changes that might occur during the study period. Finally, this design allows for ample time to assess maintenance of the intervention following withdrawal of intensive study support. Assessing maintenance is critical for interventions introduced to public health facilities, where competing priorities, limited resources, and high patient-to-provider ratios may impede the sustainment of gains made during a study.

# **INNOVATION**

To our knowledge, this study is the first in East and Southern Africa to use implementation science methods to introduce and evaluate the implementation process of single visit screen and treat approach using thermal ablation to cervical cancer prevention services. Many effective interventions in health fail to translate to routine use; this is a well-recognized gap, across numerous health domains.(57) There is very little implementation science data from LMIC to inform successful implementation of interventions that prevent CC(16). We are taking the next step in using robust IS methods, including RE-AIM, CFIR and ORIC to evaluate the implementation process of SVA-SAT+TA, engaging policy makers, health system managers and providers to disrupt the low rates of treatment for women with screen positive CC lesions. We will address the lack of access to CC prevention program services by developing a contextually fit intervention, with firm rooting in the health system and lay a foundation for sustainability.

**Use of participatory IS approaches is innovative.** *The focus of our project is to evaluate the process of implementation of an easier to use technique (TA) for prevention of CC, replacing cryotherapy, to drive sustainability and inform scale up.* We recognize that organizational change is prone to be met with resistance, more so when stakeholder engagement is limited because of “top-down” decision-making. We shall draw upon the contextual expertise of facility care workers, client perspectives and experience and managers as well as policy makers who maintain a broader macro perspective in Kenya, to plan a contextually appropriate and tailored strategy for introduction of SVA-SAT+TA. To access this local knowledge, we will employ a participatory approach to engagement, using qualitative interviews and a stakeholder workshop to allow the stakeholders to review and prioritize their preferred introduction process to best ensure commitment and sustained uptake .

**We will build a novel partnership** and engage through-out project period, with reproductive health providers and the Kenya Ministry of Health (division of reproductive health, national cancer control program), county health teams and front-line providers to facilitate successful implementation and adoption of a sustainable program. Working with the Ministry of Health will ensure that the implementation of interventions is locally-informed, addresses policymaker priorities, and provides timely, relevant information to inform decision-making. While thermal ablation is not a novel intervention medical intervention, it has only recently been endorsed by the WHO for use in low resource settings for treatment of precancerous lesions of the cervix.(14) While Zambia has introduced its use in public facilities, this is not the case for other LMICs, and the intervention is new to reproductive health facilities in central Kenya. Studying thermal ablation introduction in a second setting and gathering critical information on how it is implemented across heterogenous sites will provide important guidance for Kenya and other countries who want to take it to scale. We have planned for work to understand key bottlenecks (social, organizational, economic) that will guide policymakers and other stakeholders. This approach is highly innovative in bringing implementation science methods to cervical cancer prevention.(58)

# **STUDY OBJECTIVES**

**Objective 1:** Develop a dissemination and implementation strategy to introduce SVA-SAT+TA that effectively accounts for the heterogeneity of the client, provider, and system inputs.

**Objective 2:** Deliver the SVA-SAT+TA intervention at scale in ten reproductive health clinics and evaluate implementation using the RE-AIM framework.

**Objective 3:** Compare the cost and budget impact of SVA-SAT+TA to SVA-SAT using cryotherapy.

#

# **STUDY DESIGN**

We shall use a stepped wedge design to evaluate the introduction of thermal ablation technique for treatment of women who screen positive for precancerous lesions of the cervix to ten reproductive health facilities in Kenya, using Implementation Science framework (CFIR & RE-AIM) to examine implementation process and adoption. In addition, we will use Organizational Readiness for Implementing Change (ORIC) survey tool to assess readiness and include an end user satisfaction survey at each facility at the end of each wave.

**Study Population:**

- AIM 1:
  - 55 IDI among health care providers, policy makers and end users
- AIM 2:
  - 30 HCP in small group discussions among healthcare providers per facility conducted across implementation wave (3 - HCP per facility)
  - Approximately 30 IDI among health care managers
  - 20 IDI among screen positive women treated (2- per facility)
  - 90 providers and managers to assess ORIC
  - 360 screen-positive women enrolled to the HPV sub-study
  - 3-5 HCP per facility in small group discussions participating in Pre-barrier assessment survey at the end of each intensive implementation wave.

At each reproductive health facility, we anticipate screening 30 women per month for at least 24 months (in the clinics that activate last), for a total of >7,200 women. Estimating a 5% screen positive rate, each facility will have nine screen-positive women per wave, with an estimate 360 screen positive for data abstraction. A sub-set of 360 women will be enrolled for the HPV-sub study.

Estimated total number of study participants enrolled: Approximately 595

# **IMPLEMENTATION PLAN**:

***Aim 1:* Develop a dissemination and implementation strategy to introduce SVA-SAT+TA that effectively accounts for the heterogeneity of the client, provider, and system input*.***

We will use participatory methods to conduct qualitative interviews with clients, front-line health providers, facility managers and policy makers in the Ministry of Health to assess and address barriers and facilitators to SVA uptake and provide information on the introduction of the SVA-SAT+TA intervention through stakeholder engagement, as we have done in previous IS projects.(55) Based on informal engagement with frontline health care workers and managers in the study area, we anticipate openness to the SVA-SAT+TA intervention. This baseline engagement and guidance from the frontline health workers, clients, and other stakeholders will allow structural barriers or any other unforeseen challenges to implementation to be identified early on in the project.

In-depth qualitative interviews We will use the CFIR interview guide tool *(*[*https://cfirguide.org/wp-content/uploads/2019/08/cfirconstructs.pdf*](https://cfirguide.org/wp-content/uploads/2019/08/cfirconstructs.pdf)*)* to develop questions to explore barriers and facilitators to potential *adoption, delivery, and sustainment* of the SVA-SAT+TA intervention. Four domains (and constructs) of the original CFIR will inform both data collection and analysis: intervention characteristics (relative advantage, adaptability, complexity, cost), inner setting (tension for change, compatibility, relative advantage, readiness for implementation), characteristics of individuals (self-efficacy, knowledge and beliefs) and implementation process (planning, engaging, executing). We will also include the additional domain of “Characteristics of Systems’ to capture the intrinsic influence of the following constructs: systems architecture, external funding agent priorities, strategic policy alignment, and resource continuity in global health settings.(59) In addition, the additional constructs proposed for domains of the intervention (perceived scalability) inner setting (team characteristics, collective efficacy) and process of implementation (decision-making), will also be considered. If findings do not fall within these pre-identified constructs of interest, additional constructs will be added inductively using a grounded theory approach. We will interview frontline health providers carrying out CC screening in RH clinics (n= 30). These health providers will be purposively sampled by profession to ensure representation of nurses, clinical officers and doctors who provide reproductive health care as well as a subset with management roles (n= 10). We will also interview two female patients recently screened for cervical cancer in each study facility (n=20) and policymakers (n=5) stationed at the National Cancer Institute and the Division of Reproductive Health at the Ministry of Health of Kenya. Sample size determination in qualitative research rests on maximizing potential for saturation (when new interviews do not meaningfully add to codes and themes already represented in the previously collected data).(60) Our investigative team has extensive experience conducting in-depth interviews, group interviews and focus group discussions with purposefully-sampled health providers.(61-64)

Qualitative data analysis. Interviews will be recorded and transcribed. Transcripts will be uploaded to qualitative data management computer program Dedoose *(*[*https://www.dedoose.com/*](https://www.dedoose.com/)*)* or other qualitative software and analyzed using qualitative content analysis primarily following a deductive approach, using the CFIR as a guiding framework (as described above). Two coders will independently code facility level transcripts for each CFIR construct. Coding will be compared across the two coders and differences discussed to agree on final coding. With input from a qualitative expert, ratings that will be assigned for each construct within each facility. Ratings will be applied to reflect the positive or negative influence (valence) and the strength of each construct as described previously by Damschroder, et al.(65) Constructs will be coded as not distinguishing between potential for high and low implementation (0), or weakly (+1/-1) or strongly (+2/-2) distinguishing potential for low to high implementation performance.

**STAKEHOLDER WORKSHOP**: We will conduct a one-day stakeholder workshop, in collaboration with the National Cancer Institute to present findings from the qualitative work and introduce our study, specifically the phased introduction of SVA-SAT+TA. Invitees will include representation from the National Cancer Institute, Ministry of Health leadership, county-level reproductive health coordinators, health facility managers and select front line health providers from participating reproductive health facilities that provide VIA. At this workshop, we will invite stakeholders to suggest adaptations to the planned implementation of SVA-SAT+TA that may be necessary in specific local contexts and settings and we will attempt to incorporate ways to include these implementation guidelines for the introduction of SVA-SAT+TA.

Stakeholder meeting size will be approximately 30 – 40 persons who will be divided into three small groups to discuss determinants of implementation. To identify determinants of implementation from the workshop, the research qualitative team will summarize key points from small group deliberations and present these summary findings back to the stakeholders for consensus. We will use this feedback to refine our design to be compatible with facility workflow and systems, aligns with organizational culture and is agreeable to our stakeholders. In addition, we will use a checklist to evaluate the extent to which the deployed implementation strategies during the intensive implementation phase respond to the pre-identified barriers gathered at this stakeholder’s workshop.

***Aim 2:* Deliver the SVA-SAT+TA intervention at scale in RH clinics in Kenya and evaluate implementation using the RE-AIM framework**

Overview. At 10 reproductive health facilities in central Kenya, we will introduce the SVA-SAT + TA intervention developed and refined in Aim 1 in a stepped-wedge fashion and rigorously evaluate how effectively this strategy is disseminated and implemented. In three successive waves, with a new wave starting approximately every six months, we will roll out the intervention at 3-4 clinics per step (Fig 2). A final maintenance phase will permit evaluation of the stability of the intervention over time without direct study inputs.

**Clinic Study Setting:**

We will implement this work in ten health facilities in Embu, Kiambu, Nairobi and Murang’a counties in central Kenya, including urban faith-based , rural and urban health facilities (Table 3). Reproductive health services in the county are led by a county gynecologist and managed by reproductive health coordinator. In large health facilities, a resident gynecologist leads reproductive health services. Services in smaller health facilities are led by a clinical officer or nurse. Reproductive health services in Kenya are mostly offered by nurses. Selected facilities currently use VIA for CC screening, they screened 1237 women over a four month period with a positivity rate of 5% . This sample was purposively selected to be nationally representative and heterogeneous in terms of staffing and service.

**Table 3. Women screened for CC* in selected reproductive health facilities**

| **Facility** | **Women screened**  **(Jan-Apr 2020)** | **Screen positive** |
| --- | --- | --- |
| Kiambu level 4 hospital | 350 | 19 |
| Nyeri level 5 hospital | 206 | 8 |
| Thika level 5 hospital | 117 | 7 |
| Muranga level 5 | 45 | 4 |
| Coptic hospital (urban) | 74 | 8 |
| Karatina level 4 | 181 | 3 |
| Othaya level 4 | 91 | 10 |
| Kiandutu health center | 88 | 5 |
| Ngoliba health center | 10 | 2 |
| Ruiru level 4 | 75 | 4 |
| **Total** | **1237** | **70** |

Average of 300 screened per month and 5% positive

*Cervical cancer (CC)

**Justification for stepped wedge design**. Stepped wedge designs use sequential roll-out of an intervention (to individuals or clusters – in the present case, RH clinics) over time to assess the impact of an intervention, with the order of receiving the intervention determined at random.(56) The design is especially relevant for proven interventions that cannot be withheld ethically, but which cannot necessarily be moved to scale simultaneously in all the clinics with the resources at hand. All clusters are thus offered the intervention by the end of the study, with the order of initiation randomly assigned. Prior to a cluster offering the intervention, the cluster contributes data to the “pre-implementation” (e.g., control) period and once the cluster has begun to implement the intervention, it contributes to the “intervention” period.

This design allows for comparisons of key outcomes in the intervention versus control periods. SVA-SAT+TA is an ideal intervention candidate for a cluster**-**randomized stepped wedge design, given that it is

1) an evidence-based intervention

2) it is logistical and feasible to scale in a phased fashion (which then allows for assessment of implementation with each wave)

3) is implemented at the unit of the facility (i.e., thus we can randomize to phase of implementation at the clinic level).

This design also allows us to quantify the effect of the intervention on screening coverage and treatment effectiveness, while providing data on background changes that might occur during the study period. Finally, this design allows for ample time to assess maintenance of the intervention following withdrawal of intensive study support. Assessing maintenance is critical for interventions introduced to public health facilities, where competing priorities, limited resources, and high patient-to-provider ratios may impede the sustainment of gains made during a study.

***Procedures and Implementation strategies****:* We will implement this work in 4 phases i.e., baseline data period, training phase, intensive implementation phase and maintenance phase.

***Baseline data period****:* We will obtain baseline data on facility-level infrastructure including availability of equipment, supplies, medications as well as current staffing (numbers, cadre and training) and patient flow. Routine data on the proportion of women screened, screened positive, and treated for abnormal lesions will be abstracted monthly, by research staff, for a minimum period of 6 months prior to introduction of the intervention starting with the first wave facilities (Fig**. 3).** During this period, all clinics will contribute data to the “control” arm.

**Figure 3: Stepped Wedge Implementation**

***Randomization****:* Clinics will be randomly assigned to start intervention delivery at one of the three study steps, with clusters of 3-4 clinics per step; randomization of clinics to the order of intervention steps will be stratified by size and will take place at the stakeholder meeting, which will include participation of clinic personnel.

***Training phase****:* Before SVA-SAT + TA implementation, we shall train health providers in participating RH clinics on thermal ablation techniques, inclusive of refresher training on VIA/VILLI as part of single-visit-approach as per IARC guidelines.(66). The training curriculum will be adapted to address gaps identified during Aim 1. The research training team will provide in person, on-site hands-on training at each facility followed by technical support for a duration of one week. Each trainee will be assessed for proficiency, which will be determined through the observation of a preset number procedure and certified with a proficiency certificate. To create demand and provide an adequate number of clients for screening and demonstration of TA techniques, our team which has over 15 years of successful community engagement in this region, alongside RH clinic personnel will conduct outreach community activities prior to scheduled on-site training. The selected RH clinics have some of their providers trained on VIA/VILI and cryotherapy. Our team of investigators has three reproductive health experts with TA training skills (Leeya Pinder, Nelly Mugo, Monica Oguttu) who will train trainer of trainers at each RH clinic. Training will be provided with engagement of the KMET cure-cancer program who will provide consultant training services for the trainer of trainers for the initial 2 clinics, thereafter, the research team, led by N. Mugo, together with the county trainer of trainers will provide on-site hands-on training. N. Mugo (project-PI) and Leeya Pinder are skilled in colposcopy, loop electrosurgical excision procedure (LEEP), cryotherapy and TA, with years of experience training health care providers in addition to providing cervical cancer clinical services, will lead HCP training. In addition to thermal ablation training, quarterly continuous medical education (CME) which will include refresher CMEs on cervical cancer prevention and refresher didactic training on VIA will be provided during implementation.

***Intensive implementation phase****:* Several activities will be conducted during this phase:

1. *Technical Assistance and supervision:* We shall provide on-going technical support with assessment of fidelity to SVA-SAT +TA implementation for 6-12 months. This support will be provided by Technical assistants (research personnel) with training and expertise in conducting SVA-SAT with thermal ablation. They will visit health facilities bi-weekly to observe, mentor and provide oversight for RH health providers as they begin implementation of SVA-SAT+TA. A standardized technical assistance report will be used to document their findings, which will detail challenges faced by RH facilities which may include access to supplies, staffing retention, and any other specific requirements identified by the providers.
2. *Data Abstraction:* Individual level programmatic data will be abstracted from client record using a pre-set data collection form and will include provider information, type of treatment procedures (TA, cryotherapy, others) provided to screen positive women and clinical care outcomes related to treatment.
3. *Support supervision visits:* Quarterly, the county reproductive health coordinator or designee will accompany technical assistants to health facilities to conduct supportive supervision. Refresher trainings will be scheduled to address proficiency gaps identified during these visits.
4. *Regularly scheduled research meetings* with technical assistants and study investigators will discuss technical assistants facility level SVA-SAT+TA implementation reports and address identified challenges. This strategy for product scale up has been successfully applied in the Partners Scale Up project, led by Nelly Mugo (site PI) in 3 regions in Kenya.(54, 67) Implementation challenges will be discussed and solutions proposed.
5. *Quality assurance (QA):* Training sessions on data completion will be included to address challenges with quality of data from routine program data (e.g., incomplete, untimely data,) prior to implementation and during support supervision visits. Concerns arising from QA assessment will be reported to the regularly scheduled county and facility committee meetings and corrective action will be planned collectively.
6. *Formation of County and Facility Committees:* The county coordinator will be asked to convene a committee that will serve an advisory team at the county level, hosted by the County RH Coordinator. At the facility level, the RH team will be requested to convene an advisory sub-committee, that will work closely with the research team to provide input on project implementation processes and receive updates provided by the research team. The county and facility committees will be involved in the decision-making regarding provision of resources to support services. The research team will liaise with the committees to schedule regularly scheduled meetings.

**Maintenance phase**

At the end of implementation for the last wave of clinics, all ten clinics will concurrently commence maintenance evaluation (Fig. 3). Technical support, however, will reduce after six months of intervention implementation. RH facilities will be assessed to determine their readiness for reduced technical assistance support using predefined metrics. Metric measures will include a review of number of providers trained and implementing SVA-SAT+TA, treatment completion rate, and experience of adverse events. The assessment will determine whether intervention implementation by facility personnel without the intervention of research personnel is maintained. Bi-annual facility level assessments will be done and they will include a review of infrastructure and staffing to see if there are changes in the preparedness across sites over the maintenance period*.*

**Outcome Variable:** Our primary study outcome is change in treatment completion rate among screen-positive women, comparing pre-and post-intervention periods. The impact of this work will be evaluated using the RE-AIM framework as follows (Table 4) :

- **REACH** refers to the number, proportion or representativeness of individuals or entities reached by an intervention. We will assess the number of providers trained within each clinic. If SVA-SAT + TA is adopted and implemented as intended, we expect the number of women receiving cervical cancer screening and treatment services to increase. We will abstract individual level program data and estimate the proportion of women aged 25-49 years seeking services in reproductive health clinics who are screened per month (target = 20%) and proportion of screen-positive women who complete treatment (target = 60%) comparing pre-implementation period to intervention periods. This will include women treated with cryotherapy, LEEP or any other procedure.

**Table 4: Summary of outcomes, by RE-AIM domain**

| RE-AIM Domain | Outcomes | Data source |
| --- | --- | --- |
| REACH | - Proportion of providers trained, and women screened per month | - Abstracted client records - Facility level data |
| EFFECTIVENESS | - Number of women screen-positive per month - Treatment completion rate among screen-positive women compared to pre-intervention (Primary) - Proportion of women with clearance of HPV 6 months post receiving TA treatment | - Facility level data - HPV test results from randomly selected women |
| ADOPTION | - Proportion of selected clinics incorporating thermal ablation into routine care through updated policies - Proportion of trained providers performing SVA_SAT +TA - Facility readiness | - Facility level data, Technical assistance reports - ORIC scores |
| IMPLEMENTATION | - Core components of the SVA-SAT+TA - Description of drivers of success/failure using CFIR constructs of interest | - Facility level data, Technical assistance reports - Key informant interviews |
| MAINTENANCE | - Proportion of clinics that continue to provide and sustain SVA-SAT+TA services 12,18 and 24 months after intervention implementation | - Facility level data |

- **EFFECTIVENESS** is a clinic and individual level measure that refers to whether the intervention had intended and unintended effects. We will determine whether the intervention is associated with a change in the number of screen-positive women identified per month and a substantial increase in treatment completion rate, comparing pre-and post-intervention periods.

Through an HPV-sub study, we shall assess fidelity of implementation of TA techniques by assessing for HPV clearance. We will collect HPV cervical swab prior to and 6 months post treatment to assess for clearance of type-specific HPV infection (target 60% HPV clearance) post-treatment on an estimate of 360 screen positive women treated with TA. HPV samples will be shipped to the UW/UON HIV/STD Research laboratory in Mombasa.

**Power and sample size** According to data on cervical cancer screening and preventive treatment for Kiambu County from 2017-2019, 20% of cervical cancer screening clients who screened positive received cryotherapy or LEEP. Routine data from our proposed study facilities show an average of 30 clients per month were screened at each facility in the first three months of 2020 (**Table 3**), corresponding to approximately 10% of eligible clients. The primary effectiveness outcome is the proportion of screen-positive women who receive same day ablation treatment. Assuming six-month waves, 30 clients screened per month and a 5% screen positive rate, we will have an average of 9 screen-positive women per facility per wave. Assuming an intracluster correlation coefficient of 0.2, alpha=0.05 and 20% treatment coverage pre-intervention, we will have 80% power to detect a three-fold increase to 60% treatment coverage using thermal ablation (R (v 3.6.3) package swCRTDesign (v 3.1)). We estimate that 90% of screen positive women will be eligible for ablative treatment.(49)

**Data analysis**: The primary analysis of proportion of screen positive women who are treated pre vs post- intervention. We will use weighted least square methods appropriate for this facility-randomized three-wave step wedge design, where all clinics begin in the control condition, We will consider the intervention effect to be fixed throughout the exposed period, but random effects per facility and wave. To analyze secondary outcomes of increase in proportion of trained providers (the **REACH** outcome) and increase in number screen-positive (secondary outcome) we will use the same analysis approach for the binary and count outcome. Exploratory analysis will examine potential effect modification of facility-level factors such as patient volume, patient to provider ratio, and women screened. Potential multi-level adjustment variables include individual-level factors such as age and access to resources. The HPV sub-study in women treated with TA will assess HPV clearance in HPV-positive women. Assuming 70% of screen-positive women test positive for HPV prior to TA (N = 140), the confidence interval for HPV clearance will be +/-8.1%

- **ADOPTION**

Our primary outcomes for adoption will be

1. the proportion of clinics incorporating TA into routine care through updated policies
2. Proportion of trained providers performing SVA-SAT +TA for screen positive women and
3. health facility readiness to implement SVA-SAT+TA.

The Organizational readiness for implementing change (ORIC) tool, will be used to measure the extent to which organizational members are psychologically and behaviorally prepared to implement organizational change, such as decisions to adopt interventions like SVA-SAT+TA.

Components of readiness which will be covered within the ORIC will include

1) change commitment, reflecting shared resolve to implement a change.

2) change efficacy, reflecting a shared belief in the collective capability to implement change.

To understand readiness for adopting SVA-SAT+TA, we will apply the validated ORIC assessment scale translated and adapted to this context after each implementation wave begins (typically within 1-3 months after start, so that participants are knowledgeable of the intervention but it is still relatively new). ORIC is a 12-item Likert-type scale, broken into domains of change commitment (4 items) and change efficacy (8 items), and has demonstrated reliability, content validity, structural validity, structural invariance, and known-groups validity in field application. We will apply the ORIC to 3 management team members of each intervention facility (n=30) and 6 frontline health workers per intervention facility (n=60). Analysis will test whether sufficient inter-rater reliability and inter-rater agreement exist to aggregate individual responses to the facility level. If tests do not justify aggregation, we will use a measure of intra-facility variability in readiness rather than a facility-level mean in our analysis. The resulting analysis will provide readiness profiles for each facility as they initiate implementation, which will complement adoption, implementation and effectiveness data in understanding the broader impact of SVA-SAT+TA.

- **IMPLEMENTATION** of SVA-SAT+TA will be measured at the organizational levels to establish consensus on the core components of the intervention as well as determine the drivers of its implementation success and failure. We will use a CFIR-guided lens to review and describe SVA-SAT+TA implementation across a heterogenous sample of health facilities (described above). A participant Informed Consent Form (ICF) will be offered before the interview process where we will seek to know experiences with SVA-SAT+TA implementation.
- CFIR will guide our examination of the implementation processes and adaptations across the different facilities as well as document determinants of success and failure. We will conduct small group discussion with health providers (n = 3) at each clinic and in-depth interviews with health managers (n = approximately 5) and screen-positive women ( 2 per clinic, n=20) at spread out over the intensive implementation wave. Interviewees will be purposively selected to capture diversity in health facilities (rural vs urban, large vs small size, government facility vs mission facility). Interview guides will include questions adapted from the CFIR question bank (cfirguide.org) to gather data about selected constructs from 4 of the established CFIR domains (Table 6). Outer setting constructs of interest will be captured via fields to identify critical external events. In addition, we will also attempt to capture newly identified constructs (and one additional domain) identified by Means, et al. Questions associated with these novel constructs will be piloted for understandability and appropriateness prior to data collection. Constructs of interest may be adapted based on the outcomes of Aim 1.

**Table 5: Potential questions to providers and managers by CFIR construct**

Additionally, at the end of each intensive implementation wave, we will administer a checklist among a small group of 3-5 healthcare providers per facility. The purpose of this checklist will be to evaluate the extent to which the deployed implementation strategies respond to the pre-identified barriers (Appendix 1). We shall include an end user satisfaction survey at each facility at the end of each wave.

Analysis**:** Interviews will be recorded, transcribed and analyzed using directed content analysis following a deductive approach, using the CFIR as a guiding framework.

Thematic analysis will be applied to analyze the open responses from healthcare providers on the administered checklist. This analysis will help us understand reasons behind the pre-identified barriers were both successfully and unsuccessfully addressed categorizing them into three level i.e., successfully, partially successful, and unsuccessful.

- **MAINTENANCE** is the extent to which an intervention and the benefits it generates are sustained over time. In the first six months of implementation we will have bi-weekly on-going technical support to health facilities. For 12 months thereafter, we will reduce visits by 50% among clinics that meet metrics for transition, we will then discontinue routine technical assistance visits, but only respond to facility requests as needed. Over this time, research staff will continue to abstract individual level programmatic data. We will continue to monitor the proportion of women aged 25-49 years seeking services in reproductive health clinics who are screened per month and proportion of screen-positive women who complete treatment. In addition, we will monitor the frequency of facility committee meetings. We anticipate that facilities will continue to implement SVA-SAT+TA because we will have utilized a participatory approach that builds ownership and organizational commitment and results in implementation of an intervention that is contextually fit.

***HPV Sub-Study***

***Eligibility:*** Screen-positive women treated for pre-cancerous lesions of the cervix who are willing to be followed up for 6 months

***Visits:*** *Visits will take place on Day one of treatment with follow up at one week and 6 months post treatment.*

At enrollment, we will abstract data from medical files. Demographic and reproductive health information will be collected. HPV cervical swabs will be taken on Day 1 prior to treatment by the primary provider at the clinic.

We will obtain HPV swabs for all screened patients (+ve or -ve) and store them as we seek resources for testing. This will allow us to have quality swabs for assessment at the screen and test visit. Women who undergo ablative therapy will be asked to return a week later to make sure they are healing well. They shall be asked questions on whether they have abdominal pain, vaginal discharge or bleeding or any complication after treatment. This is routine for all women who receive treatment as part of their regular care. At 6 months post treatment, the participants will be asked to come to the clinic to be examined and to confirm that the abnormal changes are cleared. The six-month post treatment HPV test will assess clearance of HPV infection as a proxy cure and quality of implementation. HPV samples will be shipped to laboratories a Kenya based STI Laboratory, Ganjoni, Mombasa Kenya for testing. HPV results will be provided to participants to support their on-going medical care.

The shipped samples will be stored until the testing and analysis is complete. This will be done within two years of completion of all participants follow up.

At interval periods, we shall randomly sample 20 women (two per clinic) who undergo treatment for precancerous lesion with thermal ablation for an in depth interviews.

*Retention:* Women enrolled in the HPV sub-study will be requested to provide locator information, which will include an additional contact person. The study staff will call the participants one week after treatment to assess for wellbeing and an additional call to remind them of the upcoming month 6 visit.

**Aim 3: To compare the cost and budget impact of SVA-SAT+TA to SVA-SAT using cryotherapy*.***

We will quantify and compare the costs of SVA-SAT with thermal ablation to the costs of the current recommended standard practice of SVA-SAT with cryotherapy. Using micro-costing techniques, we will estimate the programmatic costs of each strategy, including implementation costs. We will use these data to estimate the cost per woman treated under each strategy, and we will incorporate reach and effectiveness outcome data (from Aim 2) in a decision analysis model to estimate and compare the costs incurred and averted. Cost and impact data will be used to estimate the budget impact of SVA-SAT with each treatment strategy. Our primary analyses will take the programmatic perspective, and secondary analyses will include costs incurred by patients to access services and duration of time used by providers to provide services. Our approach, analyses, and results reporting will align with published economic evaluation guidelines, which will maximize the transparency and generalizability of our findings to other settings.(68, 69) We hypothesize that SVA-SAT+TA will cost less per woman treated and will increase the affordability, feasibility and impact of cervical cancer prevention relative to SVA-SAT with cryotherapy.

**Table 4. Summary of cost measures and associated data sources**

| **Cost type** | **Elements** | **Data sources** |
| --- | --- | --- |
| Start-up | Training materials; training expenses (trainer fees, staff time spent in training) | Project expense reports; project staff / civil service salaries |
| Implementation | Ongoing training expenses; technical assistance support, facility committee meetings, fidelity monitoring; community outreach | Project expense reports |
| Personnel | Time/salaries of healthcare personnel, laboratory personnel, supervisors, and administrative staff | TDABC observations; staff interviews; civil service salaries |
| Patient | Transportation expenses; other out-of-pocket expenses, including childcare; time losses | Patient surveys; local average wages |
| Supplies | Consumables such as acetic acid, CO_2_ or N_2_O refrigerant gas, specula, anesthesia, equipment maintenance; transportation costs | Clinic and project expense reports; MOH records |
| Capital | Liger thermal ablator; cryotherapy machine; laboratory equipment; vehicles | Clinic and project expense reports; MOH records |
| Overhead | Utilities; clinic maintenance | Study clinic records and expense reports; staff interviews |

**Costing data collection.**

To capture heterogeneity in costs and efficiency across settings, *we will collect cost data in a representative sample of six RH clinics.* We will use an activity-based micro-costing approach to measure start-up, implementation, personnel, supply, capital, overhead, and patient costs in each clinic with standard practice (SVA-SAT with cryotherapy) and with implementation of SVA-SAT+TA (**Table 4**). Measured costs will include those incurred in the initial screening and treatment visit as well as follow-up visits. Following published protocols, we will observe clinic visits using time-driven activity-based costing (TDABC) methods to map out and quantify the time spent on each component activity.(70) We will also conduct semi-structured interviews with staff and key personnel to inform estimates of the time and costs required for provision of relevant SVA-SAT services. TDABC and interview data will be used to estimate the personnel time required for different cadres of personnel, which will be translated into costs based on average salaries. Start-up costs, ongoing implementation costs, and other program costs will be obtained from project expense reports, clinic records, Ministry of Health (MOH) records, and published literature, as in previous studies.(71-74) Patients will be asked about out-of-pocket expenses and time losses, with time losses translated into costs using data on local wages.

**Cost analysis.** We will summarize data on costs to estimate the total cost of SVA-SAT+TA per woman. Our micro-costing approach will allow us to identify and exclude costs specific to research activities, focusing only on programmatic costs. To reflect variable costs and activities in different phases of implementation of SVA-SAT+TA, we will stratify costs by initial start-up and maintenance phases. For each strategy, we will break costs down into the categories in Table 7, above, to identify the key cost drivers. Costs will be discounted at 3% per year, and we will explore alternative discounting rates of 5% and 0%.

**Budget impact analyses.** To account for differences in reach and effectiveness by treatment option, we will develop a decision tree model to estimate the expected number of CIN2+ and cervical cancer cases that would arise under a strategy of SVA-SAT+TA relative to SVA-SAT with cryotherapy given the size of the eligible patient population in Kenya. To estimate these health outcomes, we will incorporate study data on reach and effectiveness with each strategy (Aim 2) and published data on HPV natural history and progression to cancer. Costs will be estimated using study data on the cost per woman with each SVA-SAT intervention and published data of the costs of cervical cancer treatment and care. To account for variability and uncertainty in key parameters, we will conduct sensitivity analyses by jointly varying these parameters within observed or probable ranges. The model will be programmed using TreeAge Software (Williamstown, MA). Using data on current Kenya MOH expenditures, we will estimate the budget impact of the two interventions from the MOH perspective. This analysis will reflect the opportunity costs incurred by delivery of the SVA-SAT treatment strategies as well as costs averted through prevention of cervical cancer cases. Although cost-effectiveness analyses incorporating full societal costs are beyond the scope of the proposed grant, the cost and outcome data we collect can be used for such analyses in future applications.

# **LAB PROCEDURES**

We will conduct HPV DNA genotyping using the Roche Linear array assay (which can detect 37 HPV types) with a LOD of 50 copies, or equivalent PCR assay, ideally in real-time and in Kenya. HPV samples will be shipped to laboratories a Kenya based STI Laboratory ,Ganjoni, Mombasa Kenya for testing.

HPV DNA testing will be conducted using PCR. Exfoliated cell samples are digested with 20 μg/ml protease K at 37°C for one hour. DNA is isolated using the QIAamp DNA blood mini column (Qiagen, Inc., Valencia, CA) according to the manufacturer’s protocol. Two µl purified DNA (equivalent to one 250th of each sample) are amplified in 50 µl PCR reaction and 10 µL of PCR products are dotted onto nylon filters and probed with both a biotin-labeled HPV generic probe and a biotin-labeled β-globin probe. Samples negative for β-globin DNA are deemed insufficient. Specimens determined to be HPV positive by generic probe are typed using the Roche Linear Array HPV genotyping test (Roche Molecular Systems, Inc., Alameda, CA) for 37 HPV types or similar HPV DNA genotyping test.

# **DATA**

The primary goal of this project is to develop a dissemination and implementation strategy to introduce SVA-SAT+TA and deliver the intervention in the reproductive health facilities at a scale

*Data Collection*

We will use computer-based or paper based data collection forms for research variables and link to medical records data captured through standard clinic systems (e.g. cancer of the cervix screening, intervention offered for the screen positive women). All data will be maintained in a secure location. Internal quality control reports will be run on a monthly basis.

We will use CFIR interview guide tool to assess barriers and facilitators to potential adoption, delivery and sustainment of the SVA-SAT + TA intervention

Baseline data collection on proportion of screen positive women getting treatment pre-implementation will be collected at least 6 months prior to initiation of the intervention

After initiation we will collect the following information

- Proportion of women screened per month
- Proportion of trained health care providers
- Treatment completion rates for screen positive women
- Proportion of women with clearance of HPV at month six post treatment (this will be done in a sub-study with approximately 360 women)
- Proportion of clinics that have incorporated TA into routine care
- Proportion of trained providers performing SVA-SAT+TA
- Description of drivers and facilitators using CFIR constructs of interest
- Proportion of clinics that continue to provide and sustain SVA-SAT+TA services 13, 18 and 24 months after implementation phase

*Analysis*

Qualitative analyses will identify and describe key themes and explore variation within themes. Descriptive content will be inductively assembled to form explanatory accounts.

**DATA SAFETY MONITORING PLAN**

We will establish an external data monitoring committee to periodically review data on program feasibility and metrics of service delivery. The committee will include experts in programmatic provision of cervical cancer prevention services LIMC, Kenyan, and Global topic researchers, the Kenya Network of Cancer Organizations, and cervical cancer advocates. The committee will meet approximately annually with the primary study investigators and will be governed by a charter that is agreed on by all members. Members will be charged with providing suggestions to the investigators about the conduct of the programs as well as the research components. There will not be interim analyses, as that is not suitable for this study design, and thus the committee will not make recommendations about stopping the study. Reports from all reviews and committee recommendations will be provided for submission to overseeing IRBs/ECs.

# **STUDY LIMITATIONS**

**Large scope, complex intervention and broad geographic area requiring health system buy-in.** We have worked in this region and introduced implementation of PrEP, a novel HIV prevention program, successfully in 13 public HIV clinics in Central Kenya. This work required buy-in from frontline health providers and leadership at the facility, county and national level. We will leverage the long-term partnerships we have built over time between the investigators and health providers and health managers in the region while conducting HIV prevention research to assure the success of this work.

**Changing routines can be cumbersome and may generate resistance at many levels of the healthcare system.** Access to staff for training will also be dependent on number of personnel available to continue routine services during the training period, support from clinic supervisors and interest of personnel .The development of a contextually fit intervention, through a participatory approach that involves policy makers, health managers and front-line providers will foster buy-in and ownership of the work. In addition, establishment of facility level committees that will take on an advisory role and provide input on implementation processes will encourage acceptance of the project.

**Data completeness** The study will be done at the facility level and data extracted from clinic charts, therefore there is an inherent risk of incomplete data recording

**Concurrent interventions**. It is possible that other interventions would occur simultaneously to increase uptake of cervical cancer screening and treatment completion rates among those who screen positive, resulting in outcome improvements not attributable to SVA-SAT + TA. We will collect detailed baseline data and information about each clinic’s implementation process, including contextual changes and concurrent interventions that might account for change; we will conduct sensitivity analyses to model the effect of concurrent interventions.

**Contamination.** Natural communication between facilities may lead to sharing of the intervention approach and identified best practices. Though the risk of information sharing is real, we believe that the impact of this contamination will be minimal in non-intervention facilities as the application of SVA-SAT + TA requires facility specific thermal ablation equipment, and health provider training.

**STRENGTHS OF THE RESEARCH TEAM AND FOUNDATION FOR THE PROPOSED WORK**.

We have assembled a team with methodologic expertise in implementation science (Drs. Gimbel S.), qualitative research (Dr. Ngure K), costing and health economic research (Drs. Rao D. Barnabas R.) and topical expertise in reproductive health and technical experts in thermal ablation, cryotherapy procedure and cervical cancer prevention delivery globally and within Kenya (Drs. Mugo N, Pinder L., Oguttu M) and we will work with the Kenya National Cancer Control Program (Dr. Nyangasi M.)

The PHRD-KEMRI team have over decade of experience conducting clinical trials in central Kenya and have skilled team to successfully implement this project. The ICRC-UW team will provide strong fiscal, data and coordination project support. Dr. N. Mugo in conjunction with the PAP training team has conducted training camps for training RH specialists in Kenya on colposcopy and LEEP at the Kenyatta National Hospital (2000) and the Embu Level 5 hospital, (2002) where those services continue to be provided.

# **HUMAN SUBJECTS CONSIDERATIONS**

We are proposing an implementation science evaluation of a single visit screen and treat approach using thermal ablation for cervical cancer prevention services in reproductive health (RH) clinics in Kenya. This work will involve abstraction of individual level program data among women receiving reproductive health services who are eligible for cervical cancer prevention, performance of qualitative interviews, provision of technical assistance and training for health workers and HPV testing on a random sample of selected women. We will hire and train project-dedicated research staff to abstract program data, conduct technical assistance and conduct qualitative in-depth interviews with key informants. Clinical procedures will be conducted by facility RH clinical personnel. The research team in Thika, Kenya that is affiliated with the Kenya Medical Research Institute (KEMRI) is responsible for all research related activities for this project. This responsibility includes the protection of human subjects, under the direction of the site Principal Investigator (Dr. Kenneth Ngure). The investigators at the Thika research site will prepare and submit applications to the KEMRI Scientific and Ethics Review Unit detailing plans and procedures for the implementation of the study. The same protocol will also be submitted for review by the Human Subject Division Institutional Review Board at the University of Washington (UW).

Cervical cancer screening is standard of care service in Kenya. The procedures conducted on patients in the selected intervention clinics will be part of their clinical care, with introduction of thermal ablation as an alternative to cryotherapy for treatment of women with screen positive results.

Women receiving cervical cancer screening and treatment services in participating RH clinics, will be informed of the research activity, and consent will be sought from them to permit data extraction from their clinical charts. These data will not have any personal identifiers. The research team will also seek approval from participating clinics to conduct data extraction from clinical charts, these programmatic data will be part of quality improvement activities.

Working together with the clinical team at the implementing facilities, we will develop a clinic report form that supports comprehensive data collection for women receiving cervical prevention services. Research data will be extracted from these clinical records. The data on the research clinic report form will be designed to support completeness of reporting and it will reflect standard clinical care data. This form will remain in the participant records in the health facility. We will then abstract data from this record.

Data will be entered directly to a password protected tablet and uploaded to the data files at the PHRD clinical trial facility data room. The data system at the clinical trial facility are in compliance with human subject protection guidelines. There will be no name of personal identifiers abstracted from the patient clinical files.

Written informed consent will be required for (i) participants selected for HPV testing and follow up for a 6-month period to assess for HPV clearance (ii) all qualitative interviews. The informed consent process will be voluntary and without coercion. Request to participant in the study will be done within an environment that allows the prospective participant to be well informed and to understands the purpose of the study and the expected procedures involved in participation. The informed consent form will be read and explained, and those willing to consent to participate in the study will be asked to sign a paper copy of the consent form.

After obtaining informed consent, the study staff will conduct study procedures in a private area of the clinic. Eligible reproductive health clinics will be clinics that provide cervical cancer screening services, their frontline providers, clientele and managers. We will seek permission from facilities to abstract program data and individual patient demographics and medical data.

In addition, qualitative data on single visit cervical cancer screening and treatment with thermal ablation delivery process from individual and provider key informant interviews will be collected. Logs with participant identification data will be stored in a locked file cabinet and access to all electronic data will be restricted with password and encryption protection.

**Voluntariness**

Women who screen positive for precancerous lesions will be able to access TA whether or not they consent to the HPV sub study. They will be reassured that the treatment intervention will be available to them whether or not they choose to participate in the study

**Risks**

The participants in this study will be women seeking cervical cancer screening in reproductive health clinics. Maintaining the protection of the data in this population will be paramount. Throughout the course of the study, confidentiality will be preserved, and the data will be maintained in a secure location.

We will not enroll children, prisoners, or institutionalized subjects. Data obtained at the clinics will be managed by a local research data team, routinely entered into data management software, and transferred via a secure internet connection.

Risks for women undergoing cervical cancer screening may include worry or concern, regardless of the test results. Women will be counselled about the meaning of the results. Cervical cancer screening and diagnosis might cause mild physical discomfort as the procedure involves a pelvic examination. Participants in the study may be exposed to the risks and side effects of thermal ablation. The most common side effects are mild and self-limiting and include bleeding, cramping and vaginal discharge. These risks will be discussed with the participants. Project staff will track frequency of these side effects by abstracting program and clinical data.

Participants may become embarrassed, worried, or anxious when talking about their sexual practices, ways to protect against cervical cancer and other infections passed during sex, and their cervical cancer screening results. They may be worried or anxious while waiting for their test results. Getting treatment for precancerous lesions, will require abstinence from sex for at least three weeks, this has the possibility of creating disharmony between couples. We shall work with clinic health care providers to provide support counseling and messages to spouses if deemed necessary by the client. The study staff will make every effort to protect their privacy and confidentiality.

**Protection against risk**

The study team has extensive experience asking sensitive questions related to health-seeking behaviors, finances, and attitudes towards new health technologies or delivery models for prevention interventions known to be efficacious.

All research data will be labeled only with participant ID number and no identifying information will be transmitted

**Potential Benefits**

Participants will receive individualized cervical cancer risk counseling, access to cervical cancer screening and treatment of pre-cancerous cervical lesions. Women who are diagnosed with pre-cancerous lesions of the cervix will be provided with access to onsite (unless the lesion is not amenable to ablative therapy), same visit treatment.

Participants and their communities may benefit in the future from information learned from this study. The selected reproductive health clinics will receive thermal ablation equipment, personnel will be trained, provided with certificates, receive continuous medical education on cervical cancer prevention procedures and techniques during the project period. This will increase individual clinical expertise, and facilities will be more attractive to clientele due to increased access to services for women in the catchment area.

The Kenya cancer prevention and control program will receive evidence-based data to inform delivery to scale of a costed strategy that can lead the country ahead of the curve towards achieving the global agenda of cervical cancer elimination.

**Study records**

Implementation investigators will maintain, and store in a secure manner, complete, accurate, and current study records throughout the study. Study records include administrative documentation and regulatory documentation as well as documentation related to each participant enrolled, including informed consent forms, interview recordings, notations of all contacts with the participant, and all other source documents. All study records will be destroyed by the study team when records retention requirements have been met.

**Confidentiality**

Every effort will be made to protect participant privacy and confidentiality to the extent possible. Personal identifying information will be retained at the PHRD-CCR study facility and not forwarded to the University of Washington. The sites will use their standard operating procedure for confidentiality protection that reflects the input of study staff and community representatives to identify potential confidentiality issues and strategies to address them.

All study-related information will be stored securely at the PHRD-CCR clinic. All participant information will be stored in areas with limited access. Data collection, administrative forms, laboratory specimens, and other reports will be identified only by a coded number to maintain participant confidentiality. All records that contain names or other personal identifiers, such as locator forms and informed consent forms, will be stored separately from study records identified by code number. All local databases will be secured with password-protected access systems. Forms, lists, logbooks, appointment books, and any other listings that link participant ID numbers to other identifying information will be stored in a separate, locked file in an area with limited access.

**Dissemination Plan**

The study team is committed to public dissemination of results of the formative research to participants, local stakeholders and policy makers in Kenya, the global scientific community. Dissemination of study results will follow principles of good participatory practice. Results will be published in conference abstracts and peer-reviewed journals. Study results will be disseminated through presentations to local stakeholders and policymakers in Kenya, including the Ministry of Health.

# **EXPECTED APPLICATION OF RESULTS**

At the end of this project, we will have delivered the SVA-SAT+TA intervention at scale in reproductive health clinics in Kenya and evaluated implementation using the RE-AIM framework. Our findings of how effectively this strategy of SVA-SAT+TA is disseminated and implemented will inform national and county governments about the feasibility of rolling out this intervention. Qualitative interviews with health managers and health providers will shed light on the facilitators and barriers to SVA-SAT+TA intervention in public health facilities.

Cost-effectiveness data will guide policy makers’ decisions about allocation of resources towards training of health care providers and allocation of TA equipment for the prevention intervention. Finally, we will develop operational tools including training modules, clinical delivery products and assessment tools that will support delivery of this intervention at scale and will ensure that delivery continues at the national level.

# **STUDY TIMELINE**

# **REFERENCES**

1. Tsu V, Jerónimo J. Saving the World's Women from Cervical Cancer. N Engl J Med. 2016;374(26):2509-11.

2. Arbyn M, Weiderpass E, Bruni L, de Sanjosé S, Saraiya M, Ferlay J, et al. Estimates of incidence and mortality of cervical cancer in 2018: a worldwide analysis. Lancet Glob Health. 2020;8(2):e191-e203.

3. World Health Organization. A Global Strategy for elimination of cervical cancer [updated 11 September 2018. Available from: <https://www.who.int/news-room/detail/11-09-2018-who-leads-the-way-towards-the-elimination-of-cervical-cancer-as-a-public-health-concern>.

4. World Health Organization. WHO leads the way towards the elimination of cervical cancer as a public health concern (<https://www.who.int/news-room/detail/11-09-2018-who-leads-the-way-towards-the-elimination-of-cervical-cancer-as-a-public-health-concern>) 2018 [

5. Basu P, Mittal S, Bhadra Vale D, Chami Kharaji Y. Secondary prevention of cervical cancer. Best Pract Res Clin Obstet Gynaecol. 2018;47:73-85.

6. Visual inspection with acetic acid for cervical-cancer screening: test qualities in a primary-care setting. University of Zimbabwe/JHPIEGO Cervical Cancer Project. Lancet. 1999;353(9156):869-73.

7. Gaffikin L, Blumenthal PD, Emerson M, Limpaphayom K, Royal Thai College of O, Gynaecologists JCCCPG. Safety, acceptability, and feasibility of a single-visit approach to cervical-cancer prevention in rural Thailand: a demonstration project. Lancet. 2003;361(9360):814-20.

8. Sankaranarayanan R, Esmy PO, Rajkumar R, Muwonge R, Swaminathan R, Shanthakumari S, et al. Effect of visual screening on cervical cancer incidence and mortality in Tamil Nadu, India: a cluster-randomised trial. Lancet. 2007;370(9585):398-406.

9. Kenya Ministry of Health Division of Non-Communicable Diseases, Kenya National Bureau of Statistics, World Health Organization. KENYA STEPwise SURVEY FOR NON COMMUNICABLE DISEASES RISK FACTORS 2015 REPORT.

10. Ministry of Health Kenya. Kenya Health Information System(KHIS) (<https://hiskenya.org/dhis-web-commons/security/login.action>).

11. Ministry of Public Health and Sanitation and Ministry of Medical Services. National Cervical Cancer Prevention Program: Strategic Plan 2012-2015.

12. Khozaim K, Orang'o E, Christoffersen-Deb A, Itsura P, Oguda J, Muliro H, et al. Successes and challenges of establishing a cervical cancer screening and treatment program in western Kenya. Int J Gynaecol Obstet. 2014;124(1):12-8.

13. Pinder LF, Parham GP, Basu P, Muwonge R, Lucas E, Nyambe N, et al. Thermal ablation versus cryotherapy or loop excision to treat women positive for cervical precancer on visual inspection with acetic acid test: pilot phase of a randomised controlled trial. Lancet Oncol. 2020;21(1):175-84.

14. World Health Organization. WHO Guidelines for the use of thermal ablation for cervical pre-cancer lesions. World Health Organization; 2019.

15. Basu P, Meheus F, Chami Y, Hariprasad R, Zhao F, Sankaranarayanan R. Management algorithms for cervical cancer screening and precancer treatment for resource-limited settings. Int J Gynaecol Obstet. 2017;138 Suppl 1:26-32.

16. Mungo C, Osongo CO, Ambaka J, Randa MA, Omoto J, Cohen CR, et al. Safety and Acceptability of Thermal Ablation for Treatment of Human Papillomavirus Among Women Living With HIV in Western Kenya. JCO Glob Oncol. 2020;6:1024-33.

17. Randall TC, Sauvaget C, Muwonge R, Trimble EL, Jeronimo J. Worthy of further consideration: An updated meta-analysis to address the feasibility, acceptability, safety and efficacy of thermal ablation in the treatment of cervical cancer precursor lesions. Prev Med. 2019;118:81-91.

18. Ministry of Health Kenya. National Cancer Control Strategy 2017 – 2022. 2017.

19. Arbyn M, Castellsagué X, de Sanjosé S, Bruni L, Saraiya M, Bray F, et al. Worldwide burden of cervical cancer in 2008. Ann Oncol. 2011;22(12):2675-86.

20. Quinn M, Babb P, Jones J, Allen E. Effect of screening on incidence of and mortality from cancer of cervix in England: evaluation based on routinely collected statistics. Bmj. 1999;318(7188):904-8.

21. Bruni L AG, Serrano B, Mena M, Gómez D, Muñoz J, Bosch FX, de Sanjosé S. CO/IARC, Information Centre on HPV and Cancer (HPV Information Centre). HPV and Cancer (HPV Information Centre). Human Papillomavirus and Related Diseases in Africa. Summary Report. .

22. World Health Organization. Cervical Cancer: An NCD We Can Overcome (<https://www.who.int/dg/speeches/detail/cervical-cancer-an-ncd-we-can> overcome#:~:text=Cervical%20cancer%20is%20one%20of,vaccines%20are%20truly%20wonderful%20inventions.). 2018.

23. Canfell K, Kim JJ, Brisson M, Keane A, Simms KT, Caruana M, et al. Mortality impact of achieving WHO cervical cancer elimination targets: a comparative modelling analysis in 78 low-income and lower-middle-income countries. Lancet. 2020;395(10224):591-603.

24. World Health Organization. To eliminate cervical cancer in the next 100 years, implementing an effective strategy is critical. (<https://www.who.int/news-room/detail/04-02-2020-to-eliminate-cervical-cancer-in-the-next-100-years#:~:text=To%20eliminate%20cervical%20cancer%20in%20the%20next%20100%20years,an%20effective%20strategy%20is%20critical&text=Vaccination%2C%20screening%2C%20treatment%20and%20palliative,most%20common%20cancer%20in%20women>). 2020.

25. Muruka K, Nelly M, Gichuhi W, Kihara A, Eunice C, Kosgei R. Same day colposcopic examination and loop electrosurgical excision procedure (LEEP) presents minimal overtreatment and averts delay in treatment of cervical intraepithelial neoplasia in Kenyatta National Hospital, Kenya. Open Journal of Obstetrics and Gynecology. 2013;03:313-8.

26. de Sanjose S, Holme F. What is needed now for successful scale-up of screening? Papillomavirus Res. 2019;7:173-5.

27. Goldie SJ, Gaffikin L, Goldhaber-Fiebert JD, Gordillo-Tobar A, Levin C, Mahé C, et al. Cost-effectiveness of cervical-cancer screening in five developing countries. N Engl J Med. 2005;353(20):2158-68.

28. World Health Organization. Guidelines for screening and treatment of precancerous lesions for cervical cancer prevention. 2013.

29. Frederiksen ME, Lynge E, Rebolj M. What women want. Women's preferences for the management of low-grade abnormal cervical screening tests: a systematic review. Bjog. 2012;119(1):7-19.

30. Martin-Hirsch PP, Paraskevaidis E, Bryant A, Dickinson HO, Keep SL. Surgery for cervical intraepithelial neoplasia. Cochrane Database Syst Rev. 2010(6):Cd001318.

31. Sauvaget C, Muwonge R, Sankaranarayanan R. Meta-analysis of the effectiveness of cryotherapy in the treatment of cervical intraepithelial neoplasia. Int J Gynaecol Obstet. 2013;120(3):218-23.

32. WISAP Mecial Technology GmbH. WISAP Mecial Technology GmbH. Thermo Coagulation | Cervical cancer prevention | thermo-coagulation.com. Thermo Coagulation.

33. Dolman L, Sauvaget C, Muwonge R, Sankaranarayanan R. Meta-analysis of the efficacy of cold coagulation as a treatment method for cervical intraepithelial neoplasia: a systematic review. Bjog. 2014;121(8):929-42.

34. Makau-Barasa LK, Greene S, Othieno-Abinya NA, Wheeler SB, Skinner A, Bennett AV. A review of Kenya's cancer policies to improve access to cancer testing and treatment in the country. Health Res Policy Syst. 2020;18(1):2.

35. Ministry of Health Kenya. National Cancer Screening Guidelines. 2018.

36. Nyangasi M. Kenya 2019 Demographic Health Survey.

37. Ng'ang'a A, Nyangasi M, Nkonge NG, Gathitu E, Kibachio J, Gichangi P, et al. Predictors of cervical cancer screening among Kenyan women: results of a nested case-control study in a nationally representative survey. BMC Public Health. 2018;18(Suppl 3):1221.

38. Parham GP, Mwanahamuntu MH, Pfaendler KS, Sahasrabuddhe VV, Myung D, Mkumba G, et al. eC3--a modern telecommunications matrix for cervical cancer prevention in Zambia. J Low Genit Tract Dis. 2010;14(3):167-73.

39. Dykens JA, Smith JS, Demment M, Marshall E, Schuh T, Peters K, et al. Evaluating the implementation of cervical cancer screening programs in low-resource settings globally: a systematized review. Cancer Causes Control. 2020;31(5):417-29.

40. Gimbel S, Mwanza M, Nisingizwe MP, Michel C, Hirschhorn L. Improving data quality across 3 sub-Saharan African countries using the Consolidated Framework for Implementation Research (CFIR): results from the African Health Initiative. BMC Health Serv Res. 2017;17(Suppl 3):828.

41. Gimbel S, Rustagi AS, Robinson J, Kouyate S, Coutinho J, Nduati R, et al. Evaluation of a Systems Analysis and Improvement Approach to Optimize Prevention of Mother-To-Child Transmission of HIV Using the Consolidated Framework for Implementation Research. J Acquir Immune Defic Syndr. 2016;72 Suppl 2(Suppl 2):S108-16.

42. Soi C, Gimbel S, Chilundo B, Muchanga V, Matsinhe L, Sherr K. Human papillomavirus vaccine delivery in Mozambique: identification of implementation performance drivers using the Consolidated Framework for Implementation Research (CFIR). Implement Sci. 2018;13(1):151.

43. Nilsen P. Making sense of implementation theories, models and frameworks. Implement Sci. 2015;10:53.

44. Shea CM, Jacobs SR, Esserman DA, Bruce K, Weiner BJ. Organizational readiness for implementing change: a psychometric assessment of a new measure. Implement Sci. 2014;9:7.

45. Clarke GN. Improving the transition from basic efficacy research to effectiveness studies: methodological issues and procedures. J Consult Clin Psychol. 1995;63(5):718-25.

46. Glasgow RE, Vogt TM, Boles SM. Evaluating the public health impact of health promotion interventions: the RE-AIM framework. American journal of public health. 1999;89(9):1322-7.

47. Chung MH, McKenzie KP, De Vuyst H, Richardson BA, Rana F, Pamnani R, et al. Comparing Papanicolau smear, visual inspection with acetic acid and human papillomavirus cervical cancer screening methods among HIV-positive women by immune status and antiretroviral therapy. Aids. 2013;27(18):2909-19.

48. De Vuyst H, Mugo NR, Chung MH, McKenzie KP, Nyongesa-Malava E, Tenet V, et al. Prevalence and determinants of human papillomavirus infection and cervical lesions in HIV-positive women in Kenya. Br J Cancer. 2012;107(9):1624-30.

49. Greene SA, De Vuyst H, John-Stewart GC, Richardson BA, McGrath CJ, Marson KG, et al. Effect of Cryotherapy vs Loop Electrosurgical Excision Procedure on Cervical Disease Recurrence Among Women With HIV and High-Grade Cervical Lesions in Kenya: A Randomized Clinical Trial. Jama. 2019;322(16):1570-9.

50. Njagi SK, Mugo NR, Reid AJ, Satyanarayana S, Tayler-Smith K, Kizito W, et al. Prevalence and incidence of cervical intra-epithelial neoplasia among female sex workers in Korogocho, Kenya. Public Health Action. 2013;3(4):271-5.

51. Patel SJ, Mugo NR, Cohen CR, Ting J, Nguti R, Kwatampora J, et al. Multiple human papillomavirus infections and HIV seropositivity as risk factors for abnormal cervical cytology among female sex workers in Nairobi. Int J STD AIDS. 2013;24(3):221-5.

52. Zimmermann MR, Vodicka E, Babigumira JB, Okech T, Mugo N, Sakr S, et al. Cost-effectiveness of cervical cancer screening and preventative cryotherapy at an HIV treatment clinic in Kenya. Cost Eff Resour Alloc. 2017;15:13.

53. KMET. Improved Access to Cervical Cancer Screening and Treatment Options for Kisumu County Residents (<https://kmet.co.ke/news/improved-access-to-cervical-cancer-screening-and-treatment-options-for-kisumu-county-residents/>) 2020 [

54. Mugwanya KK, Irungu E, Bukusi E, Mugo NR, Odoyo J, Wamoni E, et al. Scale up of PrEP integrated in public health HIV care clinics: a protocol for a stepped-wedge cluster-randomized rollout in Kenya. Implementation science : IS. 2018;13(1):118.

55. Ortblad KF, Mogere P, Roche S, Kamolloh K, Odoyo J, Irungu E, et al. Design of a care pathway for pharmacy-based PrEP delivery in Kenya: results from a collaborative stakeholder consultation. BMC Health Serv Res. 2020;20(1):1034.

56. Hughes JP, Heagerty PJ, Xia F, Ren Y. Robust inference for the stepped wedge design. Biometrics. 2020;76(1):119-30.

57. Glasgow RE, Lichtenstein E, Marcus AC. Why don't we see more translation of health promotion research to practice? Rethinking the efficacy-to-effectiveness transition. American journal of public health. 2003;93(8):1261-7.

58. Armstrong R, Waters E, Dobbins M, Anderson L, Moore L, Petticrew M, et al. Knowledge translation strategies to improve the use of evidence in public health decision making in local government: intervention design and implementation plan. Implement Sci. 2013;8:121.

59. Means AR, Kemp CG, Gwayi-Chore MC, Gimbel S, Soi C, Sherr K, et al. Evaluating and optimizing the consolidated framework for implementation research (CFIR) for use in low- and middle-income countries: a systematic review. Implement Sci. 2020;15(1):17.

60. Boddy C. Sample size for qualitative research. Qualitative Market Research. 2016;19(4):pp. 426-32.

61. Ngure K, Baeten JM, Mugo N, Curran K, Vusha S, Heffron R, et al. My intention was a child but I was very afraid: fertility intentions and HIV risk perceptions among HIV-serodiscordant couples experiencing pregnancy in Kenya. AIDS care. 2014;26(10):1283-7.

62. Ngure K, Vusha S, Mugo N, Emmanuel-Fabula M, Ngutu M, Celum C, et al. "I never thought that it would happen ... " Experiences of HIV seroconverters among HIV-discordant partnerships in a prospective HIV prevention study in Kenya. AIDS care. 2016;28(12):1586-9.

63. Ngure K, Mugo N, Celum C, Baeten JM, Morris M, Olungah O, et al. A qualitative study of barriers to consistent condom use among HIV-1 serodiscordant couples in Kenya. AIDS care. 2012;24(4):509-16.

64. Ngure K, Heffron R, Mugo N, Irungu E, Celum C, Baeten JM. Successful increase in contraceptive uptake among Kenyan HIV-1-serodiscordant couples enrolled in an HIV-1 prevention trial. AIDS. 2009;23 Suppl 1:S89-95.

65. Damschroder LJ, Lowery JC. Evaluation of a large-scale weight management program using the consolidated framework for implementation research (CFIR). Implement Sci. 2013;8:51.

66. International Agency for Research on Cancer (IARC). Training Manuals (<https://screening.iarc.fr/manuals.php>).

67. Irungu EM, Mugwanya K, Mugo N, Bukusi E, Donnell D, Odoyo J, et al. Integrating oral preexposure prophylaxis services into public HIV care clinics in Kenya: Results form a pragmatic stepped-wedge randomized trial. *(Under review)*.

68. Drummond MF, Sculpher MJ, Torrance GW, O’Brien B J, Stoddart GL. Methods for the Economic Evaluation of Health Care Programmes. 3 ed: Oxford University Press; 2005.

69. Gold MR, Siegel JE, Russell LB, Weinstein MC. Cost-Effectiveness in Health and Medicine. New York: Oxford University Press; 1996.

70. Cidav Z, Mandell D, Pyne J, Beidas R, Curran G, Marcus S. A pragmatic method for costing implementation strategies using time-driven activity-based costing. Implement Sci. 2020;15(1):28.

71. Golovaty I, Sharma M, Van Heerden A, van Rooyen H, Baeten JM, Celum C, et al. Cost of Integrating Noncommunicable Disease Screening Into Home-Based HIV Testing and Counseling in South Africa. J Acquir Immune Defic Syndr. 2018;78(5):522-6.

72. Smith JA, Sharma M, Levin C, Baeten JM, van Rooyen H, Celum C, et al. Cost-effectiveness of community-based strategies to strengthen the continuum of HIV care in rural South Africa: a health economic modelling analysis. Lancet HIV. 2015;2(4):e159-68.

73. Ying R, Sharma M, Celum C, Baeten JM, van Rooyen H, Hughes JP, et al. Home testing and counselling to reduce HIV incidence in a generalised epidemic setting: a mathematical modelling analysis. Lancet HIV. 2016;3(6):e275-82.

74. Ying R, Sharma M, Heffron R, Celum CL, Baeten JM, Katabira E, et al. Cost-effectiveness of pre-exposure prophylaxis targeted to high-risk serodiscordant couples as a bridge to sustained ART use in Kampala, Uganda. J Int AIDS Soc. 2015;18(4 Suppl 3):20013.

**Appendix 1: Pre-identified Barrier Assessment Checklist**

The purpose of this tool is to evaluate the extent to which the deployed implementation strategies match the barriers to the SV-SAT+TA implementation identified during baseline stakeholders meeting*.*

**Instructions**

- This checklist will be administered at the end of each intensive implementation wave by study staff.
- Study staff ask each group of respondents to review the list of key barriers identified during the baseline stakeholders meeting, indicate whether the barrier applied to their facility or not and check off whether each barrier was addressed, partially addressed, or not addressed.
- Ask the respondents which implementation strategies were deployed to address each barrier. There may be more than one. (Give the participants a list of implementation strategies)
- Describe briefly any commentary on why they feel the implementation strategies were successful/ partially successful/ unsuccessful. Please insert this information in comments section.

**Participants Information**

Health facility name:

|  | PID | Gender (F/M) | Cadre (Nurse, Clinical Officer, Medical Officer, OBGYN) | Role (Health facility manager, Frontline health care worker) |
| --- | --- | --- | --- | --- |
| 1 |  |  |  |  |
| 2 |  |  |  |  |
| 3 |  |  |  |  |
| 4 |  |  |  |  |
| 5 |  |  |  |  |

|  | **Pre-identified barriers** | **Did this barrier apply to your facility (Y/N)** | **Addressed** | | | **Implementation strategies deployed** | **Comments** |
| --- | --- | --- | --- | --- | --- | --- | --- |
|  |  |  | Yes | No | Partial |  |  |
| 1 | Unstructured Clinic flow-movement of clients back and forth between registration, service delivery points and cashier's office. |  |  |  |  |  |  |
| 2 | Shortage of trained staff on cervical cancer screening and treatment (SOPs), job aids, and CC screening and treatment guidelines |  |  |  |  |  |  |
| 3 | Lack of essential medication supplies and equipment. |  |  |  |  |  |  |
| 4 | Low participants turn-out for screening. |  |  |  |  |  |  |
| 5 | Provider knowledge gap and skills on CC screening and treatment |  |  |  |  |  |  |
| 6 | High staff turnover |  |  |  |  |  |  |
| 7 | Limited space for screening and treatment |  |  |  |  |  |  |
| 8 | Cost of screening and treatment |  |  |  |  |  |  |
| 9 | Negative attitude among healthcare providers |  |  |  |  |  |  |
| 10 | Negative attitude among clients |  |  |  |  |  |  |
| 11 | Poor documentation of CC services |  |  |  |  |  |  |
| 12 | Fragmented approach to screening and treatment. |  |  |  |  |  |  |
| 13 | Stigma among CCC (Comprehensive care clinic) clients at the MCH |  |  |  |  |  |  |
| 14 | Substandard supplies especially acetic acid. (Facilities used white vinegar bought from the stores) |  |  |  |  |  |  |
| 15 | Lack of baby minders |  |  |  |  |  |  |
| 16 | Lack of participant follow-up |  |  |  |  |  |  |
| 17 | Lack of appropriate referral systems |  |  |  |  |  |  |
| 18 | Long queues |  |  |  |  |  |  |
| 19 | Inadequate privacy (screening room used for other services, students present when services are being offered) |  |  |  |  |  |  |
| 20 | Duplication of tests (VIA positive participants sent for pap smear) |  |  |  |  |  |  |
| 21 | Lack of reporting tools |  |  |  |  |  |  |

Beyond the pre-identified barriers, were there any other barriers identified during the SV-SAT+TA implementation period and subsequently addressed successfully, partially or not addressed?" (Study staff completes the table below)

|  | **Additional barriers** | **Addressed** | | | **Implementation strategies deployed.** | **Comments** |
| --- | --- | --- | --- | --- | --- | --- |
|  |  | Yes | No | Partial |  |  |
| 1 |  |  |  |  |  |  |
| 2 |  |  |  |  |  |  |
| 3 |  |  |  |  |  |  |
